# Characterization of gill bacterial microbiota in wild Arctic char (*Salvelinus alpinus*) across lakes, rivers, and bays in the Canadian Arctic ecosystems

**DOI:** 10.1128/spectrum.02943-23

**Published:** 2024-02-08

**Authors:** Flora Amill, Jeff Gauthier, Milla Rautio, Nicolas Derome

**Affiliations:** 1 Institute of Integrative and Systems Biology, Laval University, Quebec, Canada; 2 Département des sciences fondamentales, Université du Québec à Chicoutimi, Chicoutimi, Quebec, Canada; Panepistemio Thessalias Tmema Geoponias Ichthyologias kai Ydatinou Periballontos, Volos, Greece

**Keywords:** 16S rRNA gene transcripts, gill microbiota, fish, Arctic char, bacterial activity, microbial ecology, Canadian Arctic

## Abstract

Teleost gill mucus has a highly diverse microbiota, which plays an essential role in the host’s fitness and is greatly influenced by the environment. Arctic char (*Salvelinus alpinus*), a salmonid well adapted to northern conditions, faces multiple stressors in the Arctic, including water chemistry modifications, that could negatively impact the gill microbiota dynamics related to the host’s health. In the context of increasing environmental disturbances, we aimed to characterize the taxonomic distribution of transcriptionally active taxa within the bacterial gill microbiota of Arctic char in the Canadian Arctic in order to identify active bacterial composition that correlates with environmental factors. For this purpose, a total of 140 adult anadromous individuals were collected from rivers, lakes, and bays belonging to five Inuit communities located in four distinct hydrologic basins in the Canadian Arctic (Nunavut and Nunavik) during spring (May) and autumn (August). Various environmental factors were collected, including latitudes, water and air temperatures, oxygen concentration, pH, dissolved organic carbon (DOC), salinity, and chlorophyll-a concentration. The taxonomic distribution of transcriptionally active taxa within the gill microbiota was quantified by 16S rRNA gene transcripts sequencing. The results showed differential bacterial activity between the different geographical locations, explained by latitude, salinity, and, to a lesser extent, air temperature. Network analysis allowed the detection of a potential dysbiosis signature (i.e., bacterial imbalance) in fish gill microbiota from Duquet Lake in the Hudson Strait and the system Five Mile Inlet connected to the Hudson Bay, both showing the lowest alpha diversity and connectivity between taxa.

**IMPORTANCE:**

This paper aims to decipher the complex relationship between Arctic char (*Salvelinus alpinus*) and its symbiotic microbial consortium in gills. This salmonid is widespread in the Canadian Arctic and is the main protein and polyunsaturated fatty acids source for Inuit people. The influence of environmental parameters on gill microbiota in wild populations remains poorly understood. However, assessing the Arctic char’s active gill bacterial community is essential to look for potential pathogens or dysbiosis that could threaten wild populations. Here, we concluded that Arctic char gill microbiota was mainly influenced by latitude and air temperature, the latter being correlated with water temperature. In addition, a dysbiosis signature detected in gill microbiota was potentially associated with poor fish health status recorded in these disturbed environments. With those results, we hypothesized that rapid climate change and increasing anthropic activities in the Arctic might profoundly disturb Arctic char gill microbiota, affecting their survival.

## INTRODUCTION

Arctic char, *Salvelinus alpinus* (Linnaeus 1758), a salmonid well adapted to cold and oligotrophic water, has a circumpolar distribution with the widest latitudinal range in freshwater fishes. It dominates most aquatic systems and is one of the top predators in the Arctic lakes (1–5), giving it a central role in the stability of the ecosystem (6). Moreover, different sympatric forms with different life history could co-exist in the same habitat or be completely isolated. One of those ecophenotypes concerns the fish’s migration with freshwater permanent residents, small residents, and anadromous populations (4). In our data, individuals were assumed to be anadromous based on local fishermen’s knowledge. They were caught during their summer migrations from the bays to rivers and lakes in August (2018 and 2019), except for fish from Salluit, sampled in May 2019 before they migrated to the bay. Arctic char are rich in omega-3 polyunsaturated fatty acids, making them an essential source for the Indigenous communities (7) and economically important for commercial fisheries (8) and aquaculture (9). Unfortunately, this species is undermined by anthropogenic activities that lead to general and rapid lake degradation (10) with warming of surface water temperature (11). Arctic lakes are particularly impacted because of Arctic amplification, which is the fact that the rising Arctic surface temperature and the impacts due to climate change are greater in the Arctic than anywhere else in the world (12, 13). Those environmental degradations could lead to the eutrophication of oligotrophic Arctic lakes (14–19) with new inputs of nutrients and ions, salinity modifications (10, 14, 20), and decreases of dissolved oxygen in both surface and deep water (21, 22). This disturbs Arctic aquatic ecosystems, including water microbiome structure (23–27) and Arctic char physiology (19, 28, 29).

Likewise, higher water temperatures induce thermal stress in the cold-adapted *Salvelinus alpinus* (30). Warmer temperatures are correlated with increased opportunistic infections by pathogens such as *Aeromonas salmonicida*, *Ichthyophthirius multifiliis,* or *Flavobacterium columnare* (31–33). Moreover, rising temperatures may enhance the invasive capacity of southern species toward the North (34–39). These invasions threaten the local fish by introducing new parasites and pathogens for whom its immune system is naive (40–42). Those biological, physical, and chemical changes alter salmonid microbiota, including Arctic char (43–46). Microbiota is the consortium of microorganisms encompassing bacteria, archaea, protists, fungi, and viruses living in and on mucosal tissues. A balanced and symbiotic relationship between the microbiota and its host keeps both healthy. However, during stressful conditions, mucus protein composition (mucins or anti-inflammatory cytokines) is altered (47–51), the immune system activity is affected (42), and the bacterial composition, diversity, and interactions (43–46, 52) are modified. These changes favor opportunistic pathogens invasion or a shift from commensal to pathogen bacteria (53) and threaten host survival. This phenomenon is called dysbiosis (54).

In teleostean fishes, microbiota are found in skin, gut, and gill mucosal tissues. Gills are important in fish physiology since they are a semi-permeable barrier between the organism and the external environment that allows gas exchange (52). They also act as a gateway for new invasive pathogens (55) and, as guts, are a hot spot for immune molecules to respond to these attacks (55–59). Gill microbiota is therefore highly suitable for assessing the impact of both extrinsic and intrinsic factors on the integrity of Artic char physiology. However, wild Arctic char microbiota studies have focused on the skin and gut tissues in a migratory context with a salinity gradient (43, 60). Here, we report the first biogeographical study on Arctic char gill microbiota to unravel how abiotic factors might influence its bacterial composition and activity.

The taxonomic distribution of active bacteria in the Arctic char gill microbiota was characterized using a 16S rRNA gene metabarcoding approach from total RNA extracts. Sequencing 16S rRNA transcripts gives us information on the active part of the gill microbiota, while 16S rDNA sequences shed light on bacterial abundance (active or dormant) and free 16S rDNA. We quantified transcriptionally active taxa within the gill microbiota of 140 Arctic char sampled in five wild populations along a latitudinal gradient to reach this goal.

**Fig 1:**
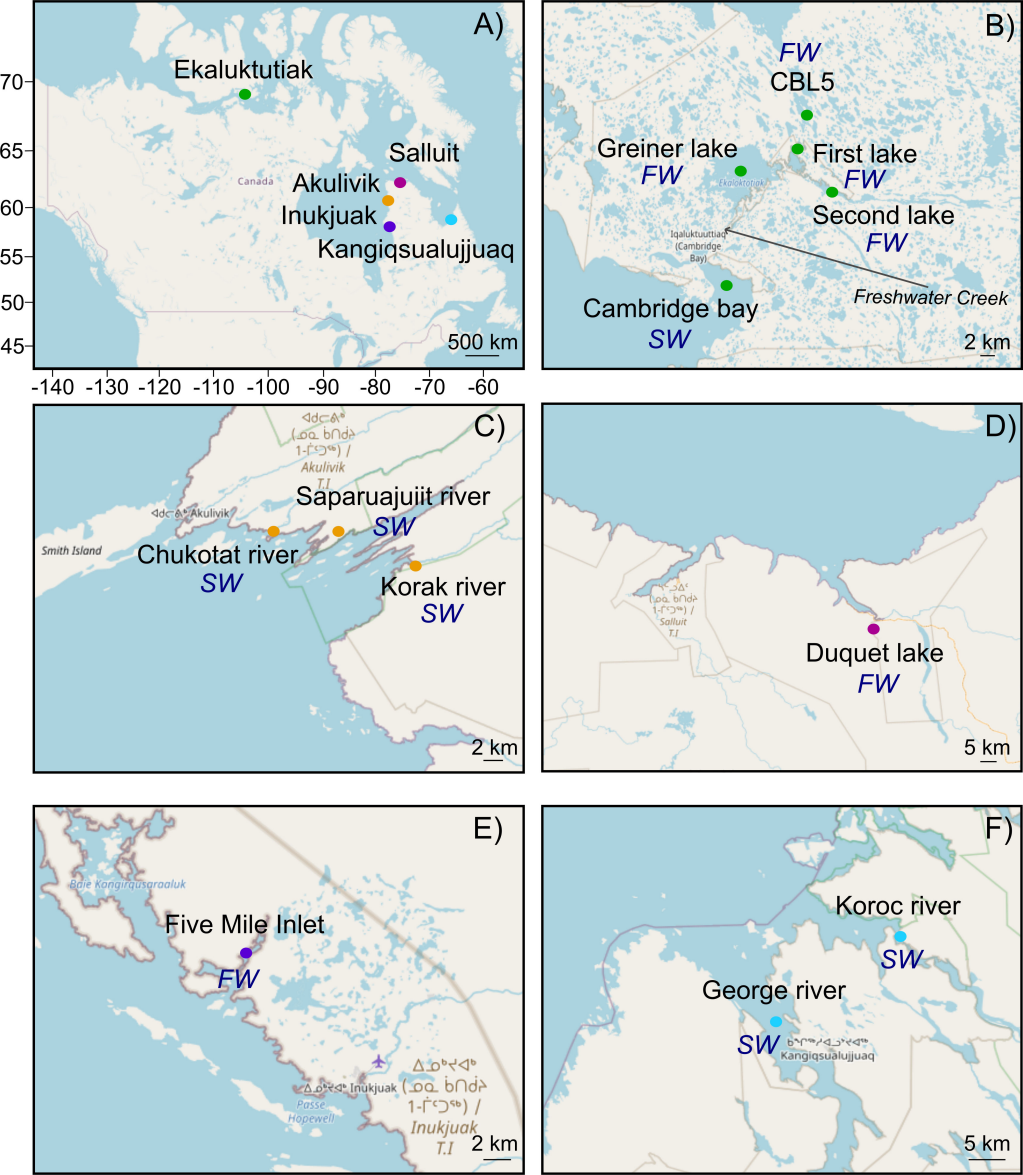
Maps of the fishing sites with the location of the main Inuit communities in Kativik, Nunavik: Hudson Strait (Salluit), Ungava Bay (Kangiqsualujjuaq), and Hudson Bay (Akulivik and Inukjuak) and in Kikmeot, Nunavut: Cambridge Bay (Ekaluktutiak) (A). Fishing sites in Ekaluktutiak were CBL5 (*n* = 7), the three connected lakes Greiner (*n* = 25), first (*n* = 12), second (*n* = 12), and the bay (*n* = 7) (**B**). In Akulivik, the three rivers Chukotat (*n* = 3), Saparuajuiit (*n* = 3), and Korak (*n* = 7) were sampled (**C**). In Salluit, Duquet Lake (*n* = 24) was the only fishing site (**D**), and in Inukjuak, the fishing site was Five Mile Inlet (*n* = 25) (**E**). Fish coming from Koroc (*n* = 5) and George (*n* = 10) rivers in Kangiqsualujjuaq were also used for this project (**F**). A total of 140 anadromous Arctic char were caught in freshwater (*FW*) and saltwater (*SW*) sites across the Canadian Arctic. Maps were created using the “leaflet” package on RStudio and manually modified in Inkscape.

We compared five different geographical sites in Nunavut (Ekaluktutiak, Victoria Island) and Nunavik (Salluit in Hudson Strait, Inukjuak and Akulivik in Hudson Bay, and Kangiqsualujjuaq in Ungava Bay) (Fig. 1) to measure the relative contribution of environmental factors (air temperature, water temperature, oxygen concentration, chlorophyll-a concentration, salinity, and pH) (Table S1) in influencing the taxonomic composition of active gill microbiota taxa. In addition, we measured the extent to which the dynamics of the interaction networks between those active taxa were modulated by these different locations. Network analysis aims to represent the complexity of active bacterial relationships according to co-occurrence patterns and positive or negative interactions between taxa (44 and references cited). This study provides a biogeographical and latitudinal baseline to monitor the gill microbiota composition and its relationship with the host’s health.

## RESULTS

The raw data showed 9,306,614 16S rRNA gene cDNA sequences with an average of 66,475 sequences per sample for 140 individuals collected from the five different communities. After filtration of low-quality reads, 8,531 amplicon sequence variants (ASVs) were available to compare gill bacterial microbiota composition between geographical sites. The abundance of the transcripts (ASVs) from active taxa differed between sites. Ekaluktutiak, Akulivik, Salluit, Inukjuak, and Kangiqsualujjuaq had 7,335, 5,380, 3,031, 4,445, and 5,751 ASVs, respectively.

### Differential relative bacterial activity between the five different sites

**Fig 2:**
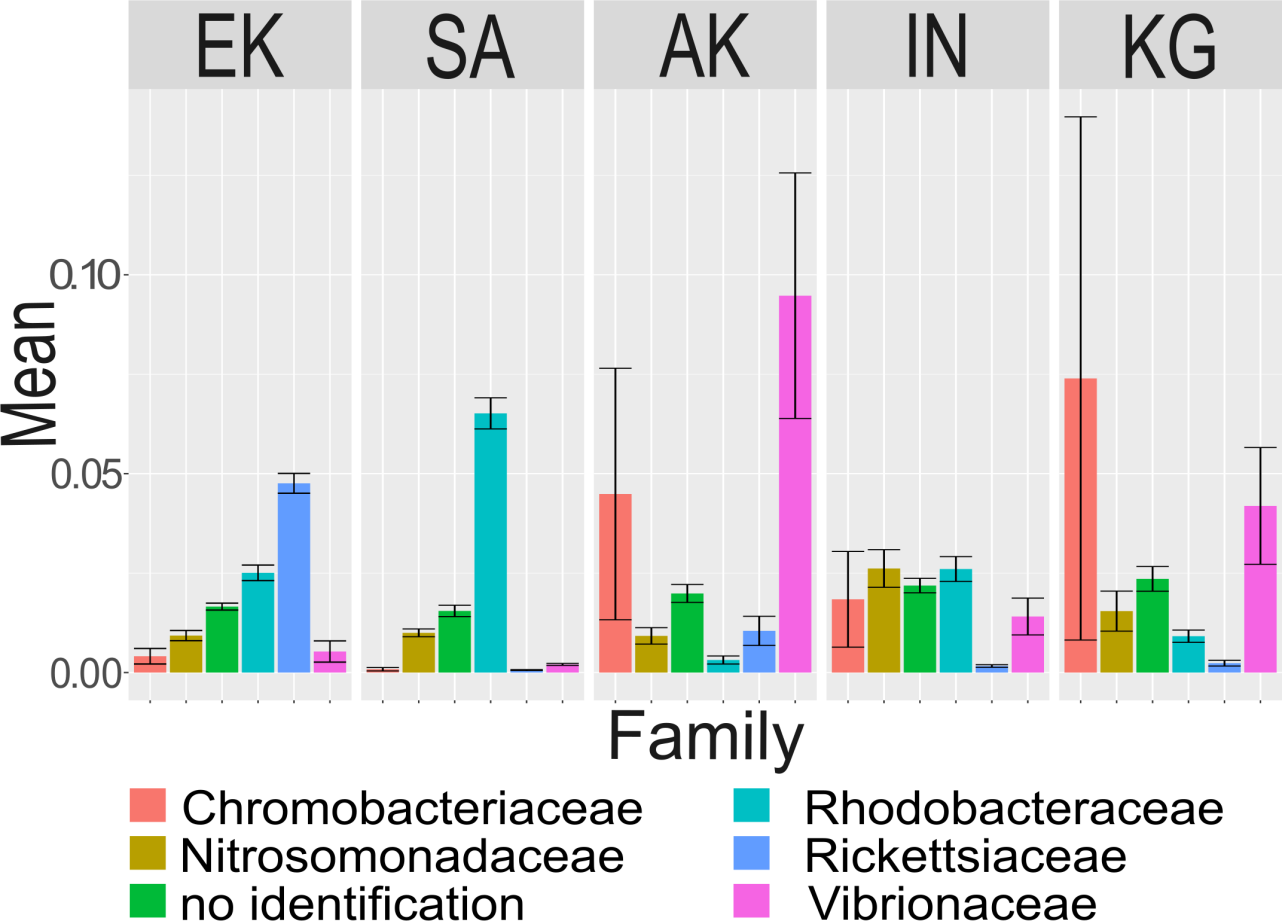
Relative activity of the 50 most abundant transcripts (ASVs) at family rank found in the microbiota of the Arctic char’s gills across the five different communities Ekaluktutiak (EK), Salluit (SA), Akulivik (AK), Inukjuak (IN), and Kangiqsualujjuaq (KG). The relative activity of ASVs in the microbiota was highly heterogeneous between sites, with different species predominating.

**Fig 3:**
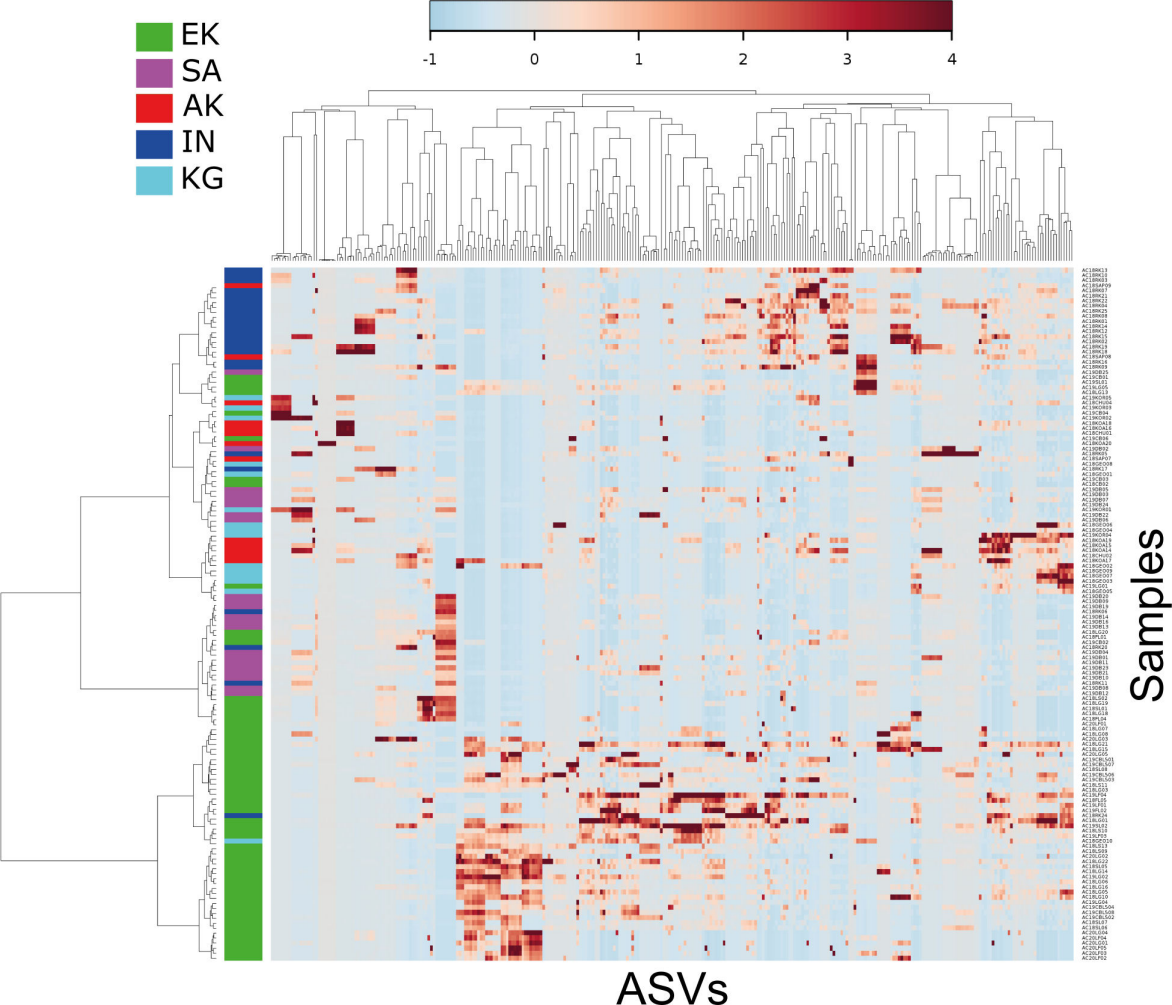
Heatmap of ASVs’ relative activity in the gills’ microbiota of Arctic char in five different communities in the Arctic. The visualization was performed with METAGENassist. In blue are transcripts with low abundance (active ASVs), and in red are transcripts with high abundance (active ASVs). The *y*-axis represents all community samples, and the *x*-axis represents all the 307 ASVs obtained after filtration. Ekaluktutiak (EK) samples were represented in green, Salluit (SA) in pink, Akulivik (AK) in red, Inukjuak (IN) in blue, and Kangiqsualujjuaq (KG) in turquoise. A pattern of an active ASV group in Ekaluktutiak showed a potentially important difference in bacterial activity in Arctic char gill microbiota between this site and the four other groups.

For the 50 most active ASVs at the family rank (Fig. 2), Arctic char gill microbiota in Ekaluktutiak was dominated by Rickettsiaceae and Rhodobacteraceae. Rickettsiaceae were significantly more active in Ekaluktutiak than in Salluit, Akulivik, Inukjuak, and Kangiqsualujjuaq (*P* = 2 e^−16^). Similarly, Rhodobacteraceae activity was significantly higher in Ekaluktutiak than in Akulivik (*P* = 1.9 e^−08^) and Kangiqsualujjuaq (*P* = 0.008) but significantly lower than in Salluit (*P* < 2 e^−16^), where Rhodobacteraceae were dominantly active. In Akulivik and Kangiqsualujjuaq, Chromobacteriaceae and Vibrionaceae transcripts were dominant but highly variable. Chromobacteriaceae were significantly more active in Akulivik than both in Ekaluktutiak and Inukjuak (*P* < 0.05), while Vibrionaceae were significantly more active in Akulivik than in Ekaluktutiak (*P* = 7.7 e^−16^), Salluit (*P* = 2 e^−05^), Inukjuak (*P =* 4.8 e^−05^), and Kangiqsualujjuaq (*P* = 0.001). Finally, Vibrionaceae activity was significantly lower in Ekaluktutiak than in Inukjuak (*P* = 8.1 e^−12^) and Kangiqsualujjuaq (*P* = 0.001) but higher than in Salluit (*P =* 1.8 e^−12^). Nitrosomonadaceae activity was also significantly lower in Ekaluktutiak than in Salluit (*P* = 1.6 e−12), Inukjuak (*P* = 2.6 e−6), and Kangiqsualujjuaq (*P* = 0.008) but higher than in Akulivik (*P* = 0.005). Also, the ASV activity heatmap (Fig. 3) showed a sample-based clustering from Nunavut, in Ekaluktutiak (in green) with an ASV group, which had an important activity compared to Nunavik samples. Another ASV group at the bottom right was also shared by Ekaluktutiak, Salluit, and Kangiqsualujjuaq samples.

### Alpha diversity

**Fig 4:**
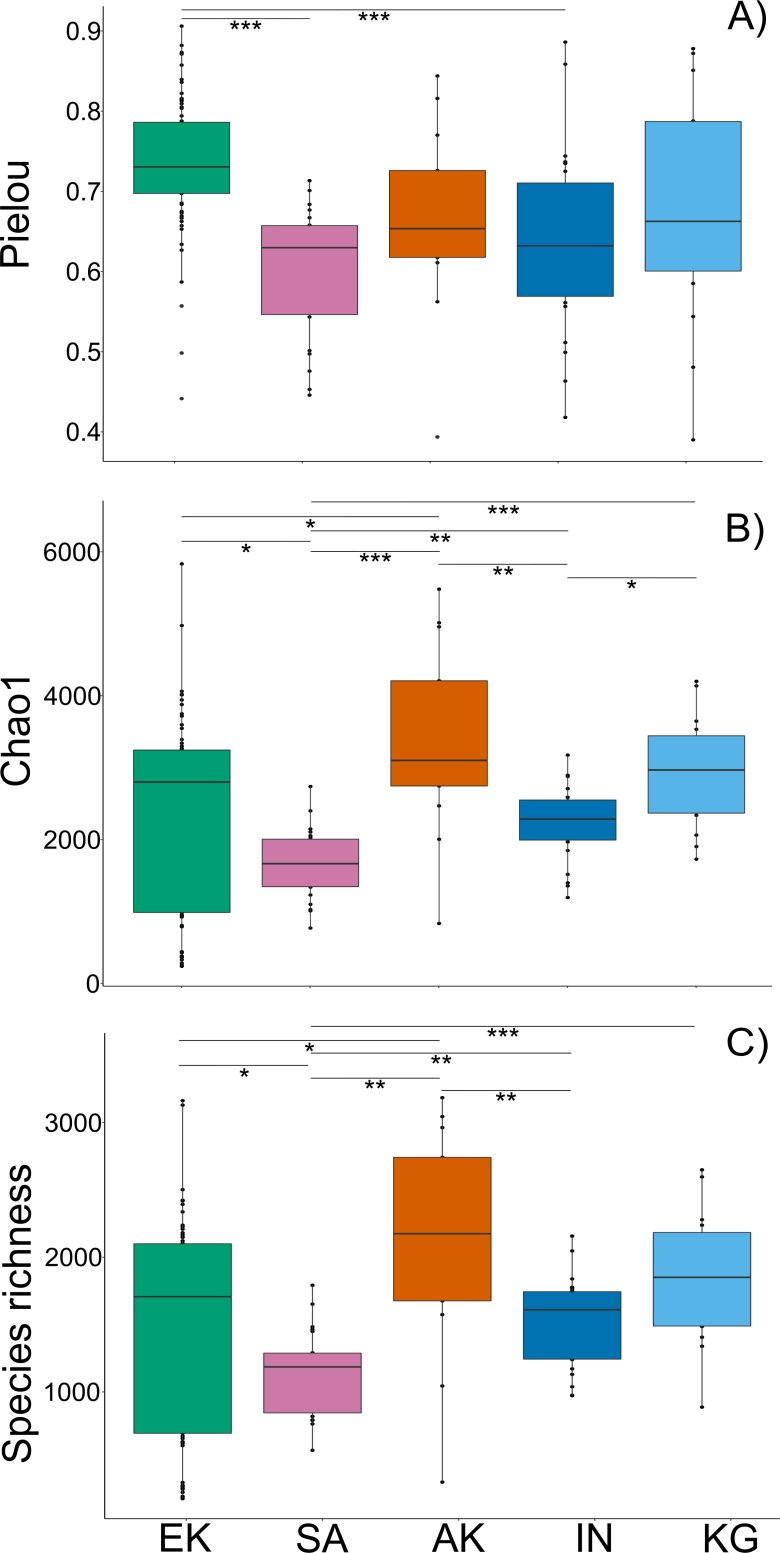
Alpha diversity. Boxplot of the Pielou index (**A**), the Chao1 index (**B**), and the species richness with Faith’s diversity index (C) across the five communities Ekaluktutiak (EK), Salluit (SA), Akulivik (AK), Inukjuak (IN), and Kangiqsualujjuaq(KG). One star indicates a significative *P*-value <0.05; two stars indicate a *P*-value <0.01, and three stars indicate a *P*-value <0.001. Sites from Salluit and Inukjuak showed the lowest bacterial alpha diversity in richness and evenness.

Pielou index calculations for alpha diversity are presented in Fig. 4A. The non-parametric Kruskal-Wallis test was performed, and significant differences between groups were found (χ^2^ = 34.24, df = 4, *P* = 6.66e−07). The pairwise comparisons using a Wilcoxon rank sum test with continuity correction showed that Pielou index in Ekaluktutiak was significantly higher than in Salluit (*P* = 4.1e−07) and Inukjuak (*P* = 0.0004). With the Chao1 estimator, a richness index that extrapolates rare taxa (Fig. 4B), we observed significant differences between groups (Kruskal-Wallis test, χ^2^ = 23.23, df = 4, *P* = 0.0001). More precisely, the Chao1 index was significantly lower in Salluit than in Ekaluktutiak (*P* = 0.04), Akulivik (*P* = 0.0001), Kangiqsualujjuaq (*P* = 3e−05), and Inukjuak (*P* = 0.002) and significantly lower in Inukjuak than in Akulivik (*P* = 0.002) and Kangiqsualujjuaq (*P* = 0.04). Faith’s phylogenetic diversity measures (Fig. 4C), which consider the overall phylogenetic distance between taxa, also showed significant differences in alpha diversity across communities (Kruskal-Wallis: χ^2^ = 20.83, df = 4, *P* = 0.0003). Salluit’s alpha diversity was significantly lower than in Ekaluktutiak (*P* = 0.04), Akulivik (*P* = 0.001), Inukjuak (*P* = 0.003), and Kangiqsualujjuaq (*P* = 0.0001), and Inukjuak showed significant lower index than in Akulivik (*P* = 0.013). Whether we use the Pielou index for evenness, the Chao1 index for richness, or Faith’s diversity index, we noticed that bacterial diversity within the Arctic char gill microbiota seemed to be the lowest in the Hudson Strait, in Salluit followed by Inukjuak.

### Geographical influence on Arctic char gills microbiota

**Fig 5:**
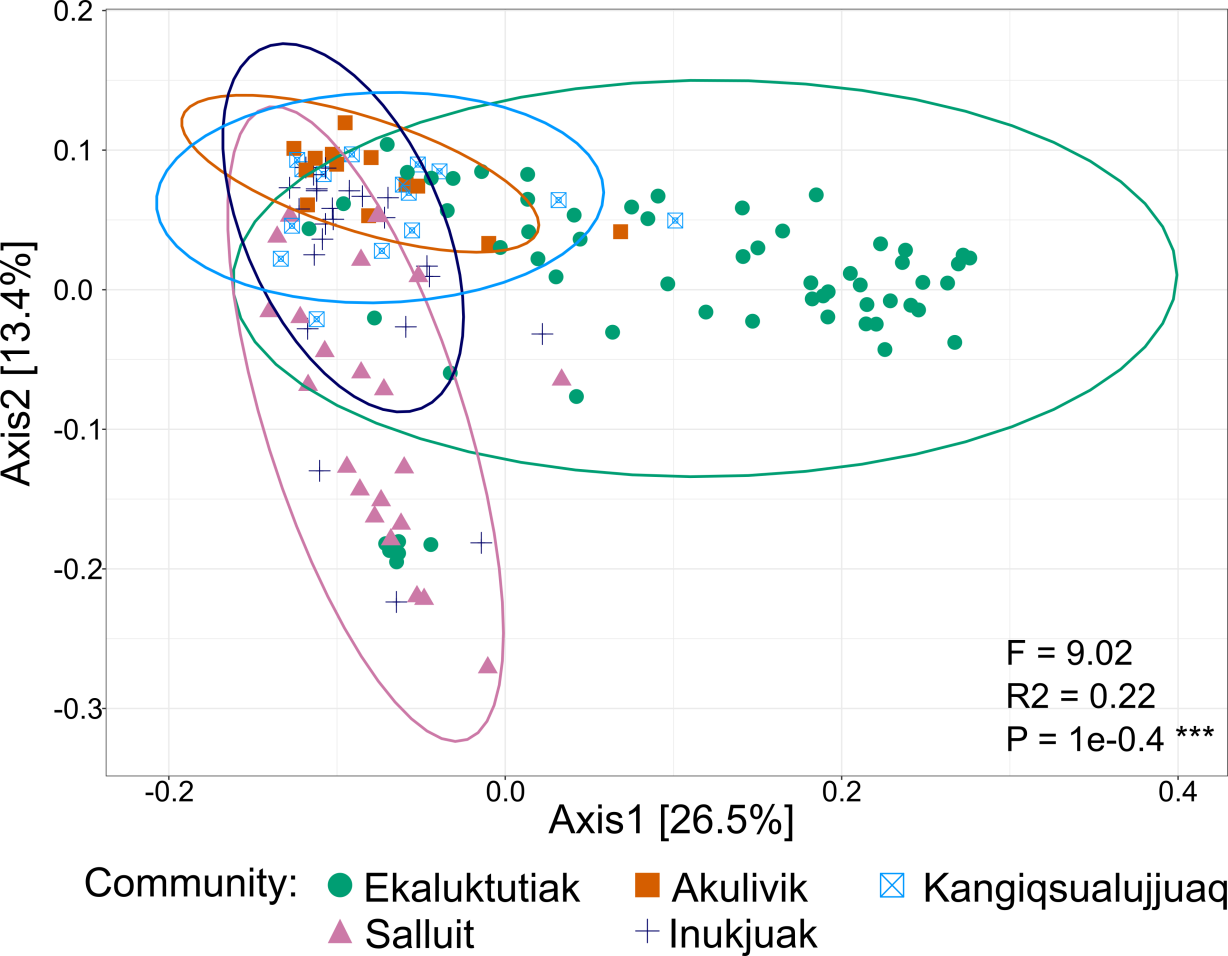
Beta diversity. Principal coordinates analysis of the samples from the five different communities in the Arctic: Ekaluktutiak (green), Salluit (pink), Akulivik (orange), Inukjuak (blue), and Kangiqsualujjuaq (turquoise). The weighted UniFrac distances were used to construct PCoA, and a multivariate analysis of variance with 9,999 permutations was performed to obtain the *P*-value. Relative ASV activity in Ekaluktutiak (axis 1) and Salluit (axis 2) significantly differed from the three other groups.

Weighted UniFrac distances between all samples from the five communities were visualized with a principal coordinates analysis (PCoA). Figure 5 shows that the geographical parameter explained 39.9% of the group variance. More precisely, Ekaluktutiak and Salluit seemed more separated from the three other groups in the first axis (26.5%) and the second axis (13.4%), respectively. Therefore, both Ekaluktutiak and Salluit gill bacterial communities appeared to be the most differentiated from the other sites. Permutation-based multivariate analysis of variance (PERMANOVA) based on weighted UniFrac distances indicated that the clustering according to geographical groups was significant (*F* = 9.02, *R*^2^ = 0.22, and *P* = 1e−04). In addition, the pairwise PERMANOVA detected highly significant compositional differentiation between groups except between Akulivik and Kangiqsualujjuaq (Table 2). Multivariate homogeneity of group dispersion (Fig. S1) showed that interindividual variation was significantly higher in Ekaluktutiak than in Salluit (*P* = 0.02) and Inukjuak (*P* = 0.01) and significantly higher in Kangiqsualujjuaq than in Salluit (*P* = 0.05) and Inukjuak (*P* = 0.01).

### Environmental influence on Arctic char gills microbiota

**Fig 6:**
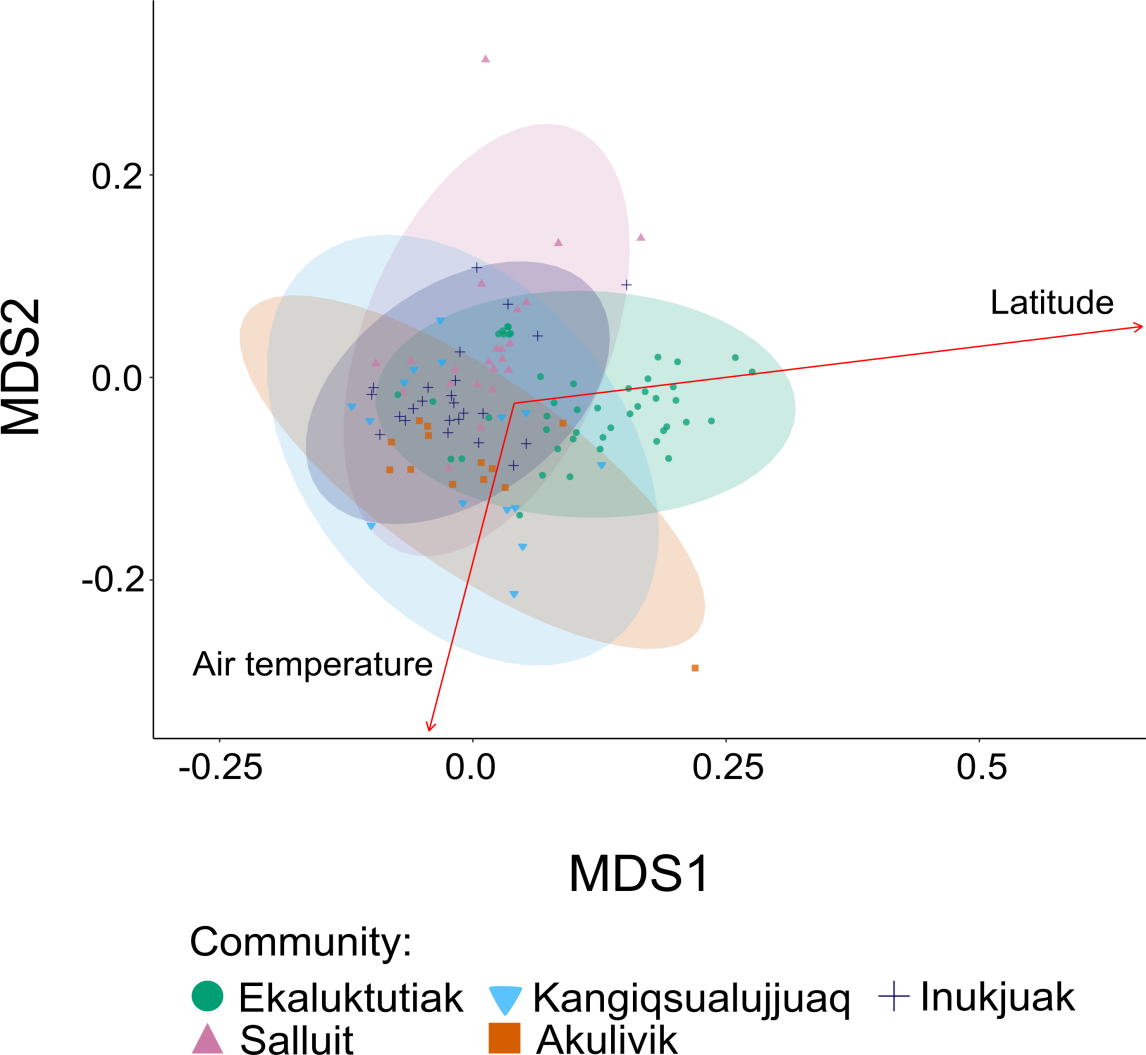
Beta diversity. NMDS on weighted UniFrac distances with environmental independent parameters fitted. The environmental parameters were represented across the samples from the five different communities in the Arctic: Ekaluktutiak (green), Salluit (pink), Akulivik (orange), Inukjuak (blue), and Kangiqsualujjuaq (turquoise). Latitude and air temperature were significantly correlated to the bacterial relative activity in Arctic char gill microbiota.

All the variables (water temperature, pH, salinity, chlorophyll-a, and O_2_ concentration) were correlated to air temperature with an absolute correlation index >0.21 (*P* < 0.05). The nonmetric multidimensional scaling (NMDS) with the factors “Air temperature” and “Latitude” (Fig. 6) showed that Ekaluktutiak was a little bit separated from the other group in the first axis of the NMDS and that latitude was significantly related to the first axis of the NMDS ordination (*P* < 0.001). In contrast, air temperature (with averages of 6.98°C, −2.35°C, 5.21°C, 7.21°C, and 15.23°C in Ekaluktutiak, Salluit, Akulivik, Inukjuak, and Kangiqsualujjuaq, respectively) was significantly associated with the second axis (*P* = 0.04). Therefore, latitude appears to be the main factor in our data set explaining the differentiation in terms of the taxonomic distribution of active bacterial strains associated with Arctic char gills between Ekaluktutiak in Nunavut and the four other geographical regions in Nunavik (Fig. S4). Given that anadromous Arctic char were sampled in various habitats such as bays, rivers, and lakes in both Nunavut and Nunavik (Table 1), the salinity effect on gill microbiota was tested both with PERMANOVA and betadisper according to the type of water: “Freshwater” or “Saltwater.” PCoA was displayed for Nunavut (Ekaluktutiak) (Fig. S5A), Nunavik (Salluit, Kangiqsualujjuaq, Inukjuak, and Akulivik) (Fig. S5B), and both regions (Fig. S5C). The effect of the type of water was significant for each separate region (Fig. S5A and B) (PERMANOVA: *P <* 0.001 and betadisper: *P >* 0.05*).* Combining, Nunavik and Nunavut samples, the water type’s effect was still significant (P < 0.001), suggesting that the type of water was another parameter explaining the different composition of the active Arctic char gill microbiota in the whole data set. Interestingly, the betadisper test had a *P*-value of 0.007, underlying the contrasting dispersion pattern between saltwater and freshwater gill microbiota (Fig. S5C).

**TABLE 1:** General information about the geographical sites Ekaluktutiak, Salluit, Akulivik, Inukjuak, and Kangiqsualujjuaq

Region	Community	Location	Type of sites	Type of water	Latitude	Longitude	Date
Nunavut	Ekaluktutiak	Greiner Lake	Lake	Freshwater	69.18	−104.99	August 2018
					69.19	−104.97	August 2019
					69.19	−104.98	August 2020
		First Lake	Lake	Freshwater	69.2	−104.76	August 2018
					69.21	−104.75	August 2019
					69.20	−104.75	August 2020
		Second Lake	Lake	Freshwater	69.18	−104.68	August 2018
					69.17	−104.6	August 2019
		CBL5 (Inuhuktok)	Lake	Freshwater	69.25	−104.71	August 2019
		Cambridge Bay	Bay	Saltwater	69.09	−105.04	August 2018
					68.99	−105.09	August 2019
Nunavik	Salluit	Duquet Lake	Lake	Freshwater	62.06	−74.53	May 2019
	Akulivik	Chukotat River	River mouth	Saltwater	60.79	−78.02	August 2018
		Saparuajuiit River	River mouth	Saltwater	60.76	−77.87	August 2018
		Korak River	River mouth	Saltwater	60.75	−77.63	August 2018
	Inukjuak	Five Mile Inlet	River	Freshwater	58.56	−78.21	August 2018
	Kangiqsualujjuaq	George River	River mouth	Saltwater	58.69	−65.95	August 2018
		Koroc River	River mouth	Saltwater	58.89	−65.79	August 2019

### Different bacteria associated with the different sites

The phylum Actinobacteria is significantly associated with the community Ekaluktutiak (IndVal = 0.70, *P* = 0.03). Fusobacteria (IndVal = 0.91, *P* = 0.001) and Firmicutes (IndVal = 0.81, *P* = 0.006) were strongly and significantly associated with the sites from Akulivik. Gemmatimonadetes was an indicator species for Inukjuak (IndVal = 0.75, *P* = 0.001). Kangiqsualujjuaq had many indicator taxa including Rhodothermaeota (IndVal = 0.86, *P* = 0.001), *Deinococcus* and *Thermus* genera (IndVal = 0.76, *P* = 0.001), Planctomycetes (IndVal = 0.69, *P* = 0.001), Chloroflexi (IndVal = 0.67, *P* = 0.001), and Nitrospirae (IndVal = 0.60, *P* = 0.005) (Table 3).

### Different dynamics in interaction networks

**Fig 7:**
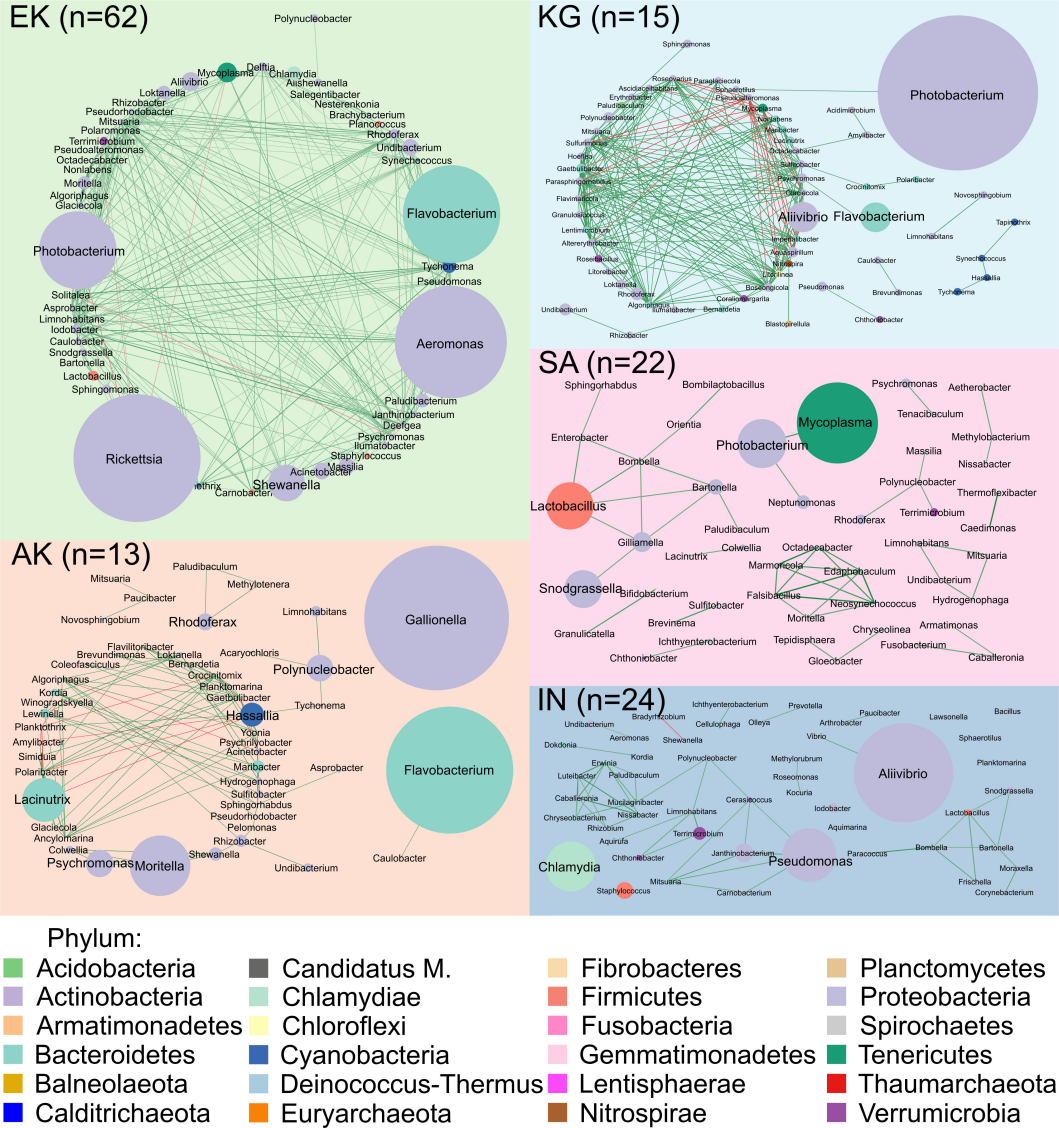
Microbial interaction networks at the five different geographic sites: Ekaluktutiak (green), Akulivik (orange), Salluit (pink), Kangiqsualujjuaq (turquoise), and Inukjuak (blue). Spearman’s correlations between the different ASVs at a genus rank, with a score <−0.4 (red edges) and >0.4 (green edges) and with a *P*-value adjusted with false discovery rate <0.05, were represented in the networks. The correlation score scales with the thickness of the edge. Each node is a genus; its size varies with its activity, and its color changes with its phylum. Ekaluktutiak was the most connected network, showing a resilient pattern, while Salluit and Inukjuak showed the lowest number of interactions.

Ekaluktutiak microbial interactions network showed 423 genera (nodes) and 8,033 interactions (edges). Contrastingly, in Salluit, only 134 nodes interacted through 288 edges. While in Akulivik, Inukjuak, and Kangiqsualujjuaq, 276, 215, and 224 nodes with 947, 826, and 995 edges were part of the network, respectively. To help in the visualization, only the 50 most active taxa at the genus rank were represented (Fig. 7). The most active taxa in Ekaluktutiak were *Pseudomonas,* with a relative transcriptional activity of 4%, followed by *Rickettsia, Aeromonas, Photobacterium* (both Proteobacteria), and *Flavobacterium* (Bacteroidetes) with relative transcriptional activities of 3%, 3%, 2%, and 2%, respectively. In Salluit, we found *Mycoplasma* (Tenericutes*), Photobacterium,* and *Lactobacillus* (Firmicutes*)* with 1% each of relative transcriptional activities. In Akulivik, *Gallionella* (Proteobacteria) with 4%, *Flavobacterium* with 3%, and *Moritella* (Proteobacteria) with 2% of relative transcriptional activities were the most active genera. In Inukjuak, *Aliivibrio* (Proteobacteria), *Pseudomonas,* and *Chlamydia* (Chlamydiae) were the most active taxa with 3%, 2%, and 2% of relative transcriptional activities, respectively. In Kangiqsualujjuaq, *Photobacterium, Aliivibrio, and Flavobacterium* were the most active genera with relative transcriptional activities of 14%, 2%, and 2%, respectively.

We observed significant differences between groups in the degree metric (DG) (χ^2^ = 122.7, df = 4, *P* < 0.001) (Fig. S6A), the neighborhood closeness (NC) metric (χ^2^ = 64.60, df = 4, *P* < 0.001) (Fig. S6C), and the connectivity centrality (CC) of the nodes in the networks (χ^2^ = 64.60, df = 4, *P* < 0.001) (Fig. S6B). Ekaluktutiak had the most connected interaction web with DG and NC, significantly higher than for Salluit (*P* < 0.001), Akulivik (*P* < 0.001), Inukjuak (*P* < 0.001), and Kangiqsualujjuaq (*P* < 0.001). CC was also significantly higher than Kangiqsualujjuaq (*P* < 0.001), Akulivik (*P* < 0.001), and Inukjuak (*P* < 0.001) but not significantly different than Salluit (*P* = 0.18). Then, Kangiqsualujjuaq seemed the most connected network after Ekaluktutiak’s with DG (*P* < 0.001), NC (*P* < 0.001), and CC (*P* < 0.001) significantly higher than Inukjuak. Kangiqsualujjuaq also had significantly higher NC (*P* = 0.05) and CC (*P* = 0.0002) than Akulivik, as well as significantly higher DG (*P* < 0.001) and NC (*P* < 0.001) than in Salluit. Akulivik had significantly higher DG and NC than Inukjuak (*P* < 0.001) and Salluit (*P* < 0.001) and significantly higher CC than Inukjuak (*P* = 0.03). Finally, Inukjuak and Salluit did not differ significantly from each other in DG and NC, but Salluit had significantly higher CC than Kangiqsualujjuaq (*P* = 0.05), Akulivik (*P* = 0.001), and Inukjuak (*P* = 0.001).

Regarding the correlations between all genera, we obtained 58, 0, 17, 2, and 43 negative correlations with 7,975, 288, 930, 824, and 952 positive correlations for Ekaluktutiak, Salluit, Akulivik, Inukjuak, and Kangiqsualujjuaq, respectively (Table S3A). Less than 4% of the correlations were negative for all the networks. In Ekaluktutiak, *Psychromonas* and *Rickettsia* were the most negatively correlated genera in the network, with five edges each (Table S3B). *Rickettsia* activity was exceptionally high (88, 166), and the topological metrics were relatively high for both genera. In Akulivik, *Acinetobacter*, *Hassalia, and Planktothrix* had the most negative correlations, with three, four, and five negative correlations, respectively. They were not the most active taxa and did not seem to be essential nodes in the network dynamics regarding the topological parameters (Table S3C). In Kangiqsualujjuaq, *Pseudoalteromonas* with nine negative correlations and *Mycoplasma* with eight negative correlations had metrics showing an important role of those taxa in this interaction network (Table S3D). Finally, Inukjuak only showed two nodes that have one negative relationship each: *Shewanella* and *Bradyrhizobium.* The nodes had low activity, and according to the metrics, they did not have a central role in the web (Table S3E). In all interaction networks, Proteobacteria and Bacteroidetes were predominant. Only a few taxa were shared between them, but they did not seem to have the same impact on the different network’s topologies. Moreover, none of the genera harboring negative correlations were simultaneously present in the different interaction networks. If Ekaluktutiak, Kangiqsualujjuaq, and Akulivik had important connectivity inside the interaction network, Salluit and Inukjuak were more fragmented (e.g., split into subnetworks), and their nodes were less connected.

## DISCUSSION

In the present study, we have characterized the bacterial composition of active gill microbiota in Arctic char at five different locations in the Arctic: Ekaluktutiak (Cambridge Bay, Nunavut), Salluit (Hudson Strait, Nunavik), Akulivik, Inukjuak (Hudson Bay, Nunavik), and Kangiqsualujjuaq (Ungava Bay, Nunavik). Arctic char gill microbiota composition was generally heterogeneous. First, we observed that the northernmost site had the most different bacterial composition from the four other sites and showed a bacterial community harboring a strong resilience pattern with high connectivity in its interaction network. In contrast, gill bacterial activity at Salluit and Inukjuak sites, heavily impacted by anthropogenic activities (61–66), showed a potential signature of dysbiosis, coinciding with reports of disrupted reproduction, infections, or increased mortality in Arctic char (67, 68). Finally, latitude and, to a lesser extent, air temperatures and water type were the most important influences in the composition of the active part of the Arctic char gill microbiota.

### Arctic char gill bacterial microbiota activity

Proteobacteria, Bacteroidetes, and Firmicutes were dominant in terms of activity. Those phyla have already been found to be major players in Arctic char gut microbiota in wild populations from Norwegian lakes (69) and experimental conditions under different diets in Sweden (70). Similarly, Arctic char core skin microbiota in the Kitikmeot region encompassed Proteobacteria, Firmicutes, and Cyanobacteria (43). When compared to other salmonids, Proteobacteria and Bacteroidetes were found in the gill microbiota (71), while Proteobacteria, Firmicutes, and Actinobacteria were found in rainbow trout gut microbiota (*Oncorhynchus mykiss*) (72–74). Proteobacteria were also found as a main phylum in Brook char (*Salvelinus fontinalis)* skin mucus with the phylum Bacteroidetes (44) and as core taxa in Atlantic salmon (*Salmo salar*) gut microbiota with the phylum Tenericutes (75) and Firmicutes (76, 77). Overall, the gill composition in teleostean fish consists of Proteobacteria, Firmicutes, Actinobacteria, Cyanobacteria, Ascomycota, and Basidiomycota (78). Therefore, our study confirmed that Proteobacteria, Bacteroidetes, and Firmicutes are part of the core microbiota of Arctic char gills. At family rank, gill bacterial communities were dominated by Chromobacteriaceae and Vibrionaceae in Akulivik and Kangiqsualujjuaq, by Rhodobacteraceae in Salluit and Ekaluktutiak, and by Rickettsiaceae in Ekaluktutiak only (Fig. 2). Vibrionaceae, a core taxon in skin and gut microbiota in wild Arctic char from King William Island (Nunavut) (43, 60), is common in marine environment (79), and Rhodobacteraceae was found in the gut microbiota of rainbow trout (*Oncorhynchus mykiss)* (80). Interestingly, Chromobacteriaceae and Rickettsiaceae are not commonly found in salmonid microbiota.

The principal genera found in the Arctic char gill microbiota were common members of teleost microbiota or opportunistic pathogens. First, *Photobacterium,* a common bacterium in marine environments (79), Atlantic salmon (75), and Arctic char skin and gut microbiota (43, 60), was found as one of the most active genera in the five communities (Fig. 7; Fig. S7A). Strains belonging to this genus could induce either benefits for the host with antimicrobial, antifungal, or antiprotozoal molecules production (81) or negative effects with pathogenic strains such as *P. damselae*, which could cause pasteurellosis, a bacterial septicemia, in marine fish (82). In Akulivik, Inukjuak, and Kangiqsualujjuaq, *Paludibacterium,* usually found in freshwater with low salt tolerance (83), was dominant (Fig. S7A). However, gills were in contact with the saline environment during migration, so the taxon found should be a salt-tolerant strain or a core taxon recruited before the migration. In Akulivik and Inukjuak, Arctic char gills also carried *Aliivibrio* (Fig. S7A). *Aliivibrio* predominates in adult Atlantic salmon gut microbiota during its marine migratory phase (75) and comprises major fish pathogens associated with dysbiosis and diseases (84). *Staphylococcu*s and *Flavobacterium*, two genera documented to include opportunistic pathogens, were dominant in Akulivik and Salluit, respectively (Fig. S7A). For example, *Staphylococcu*s *aureus* could trigger exophthalmia and septicemia (85), and *Staphylococcus warneri* could induce an inflammatory response during dysbiosis (86). Moreover, *Flavobacterium columnare* and *Flavobacterium psychrophilum* are well known in aquaculture to cause bacterial diseases, gill lesions, ulcers (87), or cold-water disease (88), including in Arctic char (53). However, commensal strains of *Staphylococcu*s were observed in rainbow trout *(Oncorhynchus mykiss)* skin microbiota, and *Flavobacterium* is considered a common member of the salmonid microbiota, as observed in brook char skin mucus (89), rainbow trout gills (71), and Arctic char gut and skin (43, 69). Finally, Ekaluktutiak showed a different profile dominated by *Aeromonas, Pseudomonas,* and *Rickettsia* (Fig. S7A). *Aeromonas* and *Pseudomonas* are two genera documented to include opportunistic pathogens triggering infection in stressed teleost hosts (44, 53, 90–93). However, Pseudomonadaceae is part of Arctic char skin and gut core microbiota (43), and *Pseudomonas* is active in many environments (94) and commonly found in the Arctic (Svalbard) (95). *Rickettsia* genus includes pathogenic and toxic species that can be transmitted to humans through ticks (96), but few reports of fish infections were found. However, a “rickettsia-like organism,” *Piscirickettsia salmonis,* is responsible for a salmonid disease: the piscirickettsiosis (septicemia), which was documented in rainbow trout (*O. mykiss*), Chinook (*O. tshawytscha*), Coho (*O. kisutch*), Atlantic (*Salmo salar),* and pink (*O. gorbuscha*) salmons (97).

### Biogeographical influence on Arctic char gill microbiota

The ASV relative activity heatmap showed an interesting pattern among the five different geographical sites, isolating the northern site in Nunavut (Fig. 3). This pattern is further supported by PCoA (Fig. 5) and PERMANOVA, showing that bacterial compositions of Ekaluktutiak and Salluit are significantly different from the three other groups (Table 2). Environmental conditions may partly drive this clustering. Indeed, microbiota taxonomic diversity and functionality are influenced by environmental parameters (43, 44, 52, 98–104), which vary between different geographical sites (98). In our study, samples were collected in four different hydrologic basins: Ekaluktutiak, located in Cambridge Bay, in the Nunavut region, whereas southeast samples were taken in Hudson Strait, Hudson Bay, and Ungava Bay, which delimit the North, the West, and the East of Nunavik, respectively. Moreover, anadromous Arctic char came from lakes (Ekaluktutiak, Salluit), rivers (Inukjuak), or from the mouth of the bay (Kangiqsualujjuaq, Akulivik, and Ekaluktutiak) at different latitudes explaining that parameters such as temperature, salinity, and productivity were different between sites (105, 106). Therefore, the different types of sites located at different latitudes with different environmental conditions could explain the significant difference in Arctic char gill microbiota regarding the taxonomic distribution of transcriptional activity. These results were consistent with previous Arctic char skin and gut microbiota studies, where the geographical sites influenced the bacterial composition and diversity (60, 98). Gill microbiota could also be influenced by the population’s genetic structure (52), and a study on the genetic populations of Arctic char in Hudson Strait, Hudson Bay, and Ungava Bay showed distinct genetic populations (106). Investigating how population genetic structure influences the Arctic char gill microbiota in our data will be interesting.

**TABLE 2:** Pairwise PERMANOVA *P*-values for the unweighted UniFrac distances with 9,999 permutations^*a*^

Unweighted UniFrac distances					
	Ekaluktutiak	Salluit	Akulivik	Inukjuak	Kangiqsualujjuaq
Ekaluktutiak					
Salluit	0.0001				
Akulivik	0.0001	0.0001			
Inukjuak	0.0001	0.0001	0.0002		
Kangiqsualujjuaq	0.0001	0.0001	0.34	0.0004	

^*a*^ *P*-values were adjusted with the Benjamini-Hochberg correction.

### Latitude and air temperature influences in Arctic char gill microbiota

The latitude had the strongest effect on the taxonomic distribution of active bacterial strains in Arctic char gill microbiota (*P* < 0.001) to explain the differentiation between Ekaluktutiak (Nunavut) and the four other groups from Nunavik. Previously, Arctic char gut and skin microbiota analyses had shown the impact of habitat, season, geographical sites, salinity, and age on bacterial composition (43, 60, 98). Here, the latitude was the main explaining factor, to a lesser extent, air temperature (Fig. 6) and salinity (Fig. S5). At first sight, water temperature was identified as one of the main factors explaining the differences in gill microbiota’s richness (diversity) and evenness (structure) across the five geographical sites (Fig. S2). However, given that we were not able to directly measure water temperature in each sampling site due to logistic constraints and different sampling teams, half of the water temperature data were obtained from GIS (geographic information system) estimation (all Nunavik samples), while the other half being directly measured with RBR probe (Nunavut). Therefore, a methodological bias was induced, potentially enhancing observed differences between Nunavut and Nunavik samples (Fig. S3). Therefore, to avoid any statistic bias, and because GIS estimation was not available for Nunavut sampling sites, we used air temperature collected by the official Canadian Data of Environment and Climate Change Canada (https://climate.weather.gc.ca/) for each Inuit community. Indeed, air temperature is generally used to calculate the water temperature as it has a strong positive correlation with it (107). The results showed a significant effect of air temperature on the taxonomic distribution of bacterial activity in Arctic char gills (Fig. 6) (*P =* 0.04). This effect was unsurprising as the temperature is one of the main factors influencing the microbial composition in waters and fish microbiota, including Arctic char (60). As an ectotherm living in cold, oxygenated, and oligotrophic waters (108, 109), Arctic char is one of the least resistant to high temperatures, thus strongly limiting its latitudinal distribution (2, 4, 30, 110). Arctic char generally live within a narrow range of water temperatures between 5.8°C and 11.4°C in freshwaters (111) and between 5°C and 8°C in saltwater (112). A temperature optimum of 9.4°C for growth was recorded in Frobisher Bay, Nunavut (113), and a lethal temperature of 18°C has been determined in controlled conditions (114). From the microbial point of view, thermal acclimatization is explained in terms of metagenomic plasticity (115). Psychrophilic bacteria adapted for low temperatures will be replaced or dominated in warmer temperatures by mesophilic strains adapted to higher temperatures and providing similar functions. In farmed Atlantic salmon (*Salmo salar)* gut microbiota, or in Chinook salmon (*Oncorhynchus tshawtscha*) gut microbiota, studied in recirculating aquaculture systems, warmer temperatures lead to the increase of the mesophilic genera *Vibrio* (116, 117) and *Brevinema* spp. (118), respectively. Moreover, a decrease in the psychrophilic *Clostridium* spp*.* in Chinook salmon gut microbiota was also noted (119). Additionally, mesophilic strains that replace psychrophilic species could be pathogenic. For example, *Aeromonas salmonicida* spp*.,* which are primarily well known for being psychrophilic (32, 120, 121), also contain many mesophilic strains (122), and its abundance was correlated with warmer temperatures and high incidence of furunculosis in fish in James Bay (Nunavik) (31). Thus, warmer water temperatures could lead to more fish diseases in the North. We found that Arctic char gill microbiota from Ekaluktutiak, with warmer water temperatures, exhibited increased activity of the mesophilic species *Aeromonas lacus, Aeromonas sobria*, and *Pseudomonas brenneri* (Fig. S7B) (123–126)*. Aeromonas lacus* was also found dominant in Duquet Lake (Salluit). Contrastingly, in colder water from Kangiqsualujjuaq, Inukjuak, and Akulivik, bacterial activity was dominated by the psychrophilic species *Aliivibrio sifiae* and the *Photobacterium carnosum* (127–129). This suggests mesophilic species dominated warmer sites, and psychrophilic species dominated colder sites. Arctic char gill microbiota in the five communities studied here was generally dominated by active genera, such as *Photobacterium, Aliivibrio, Staphylococcus, Aeromonas, Pseudomonas,* or *Flavobacterium,* which are known to include opportunistic pathogen species. Those genera could trigger infections in the fish under stressed conditions such as warmer temperatures (44, 53, 82, 84–88, 90–92). Therefore, the change in water temperatures and its impact on replacement by mesophilic opportunistic pathogens must be closely examined. More investigations with controlled environmental and ecotoxicological parameters are needed to test which parameters in those different latitudes may most influence the Arctic char gill microbiota.

### Different bacterial network dynamics and potential dysbiosis

Ekaluktutiak exhibited the most connected interacting network, involving 8,033 interactions between the 50 most active taxa. High network connectivity is a hallmark of microbiota resilience (130–133), so such results might indicate a healthy fish population. Interestingly, Arctic char stock and survival in Ekaluktutiak were relatively stable (112, 134, 135). Thus, Ekaluktutiak gills microbiota exhibited a resilient pattern that involved mainly active strains belonging to Proteobacteria and Bacteroidetes (Table S3A; Fig. 7). It is also associated with Actinobacteria, usually found in healthy salmonid microbiota (Table 3) and containing species able to synthesize antibiotic products (136), inhibiting fungal pathogen development (71), and/or producing potential probiotics for treating furunculosis in rainbow trout (58, 137). In contrast, the Salluit network exhibited the lowest number of interactions (*n* = 288) between 48 taxa (Fig. 7), involving mainly *Mycoplasma,* a genus including several opportunistic and pathogenic species (138). *Mycoplasma* is particularly abundant in salmonid gut (71, 75, 139), playing a role in lipid and sugar metabolism (140), and is a core genus in Arctic char gut microbiota in freshwaters (43, 60). Overall, with both the lowest bacterial alpha diversity, in terms of richness and evenness (Fig. 4), and the weakest network connectivity, Salluit microbiota probably showed a pattern of a highly unstable bacterial community (i.e., with low resilience) (133). Stressful conditions could induce low resilience of the gill microbiota, favoring pathogenic infections of fish (131, 132, 141–146). Interestingly, metal contaminations by the Raglan mine, located approximately 100 km upstream of Deception Bay in Salluit, have been reported (66–68, 147), and a study showed Arctic char muscular infections by fungi in this region (67). However, we cannot exclude that the difference between Ekaluktutiak and Salluit, particularly in the number of interactions between the different genera, could also be influenced by the fact that we have more sampling sites in Ekaluktutiak (five) than in Salluit (one). The low connectivity of the network and pre-dominance of the genus *Aliivibrio* suggest that the Inukjuak microbiota was susceptible to dysbiosis but to a lesser extent than Salluit. Moreover, Gemmatimonadetes (Table 3) is associated with Arctic char gill microbiota in Inukjuak. It contains a lot of phototrophic species found in wastewater treatment or the High-Arctic in Greenland (148). Many environmental stresses in Inukjuak, such as chemical contamination (69, 70), hydroelectric dams, or fish invasions bringing new parasites and pathogens (66), could cause this potential dysbiosis and threaten Arctic char. Moreover, in 2018, in the Five Mile Inlet system in Inukjuak, the Arctic char local population had a healthy Fulton index (1.13 + −0.10) but showed disruption in reproduction and a worrying annual mortality rate (68%–82%) (68). Further investigation should focus on the link between Arctic char gill microbiota, the host’s health and immune system, and more accurate environmental analysis in Salluit and Inukjuak. Akulivik and Kangiqsualujjuaq individuals exhibited healthy gill microbiota patterns, but a complete report from Makivik Corporation (66) showed important Arctic char mortality in rivers from Kangiqsualujjuaq. However, the air temperature collected in August 2019 was exceptionally high (18.86°C) (ECCC) and decoupled from the water temperature collected by GIS (2.08°C). This can be explained by the glaciers around Kangiqsualujjuaq, which also play an essential role in the water temperature due to their erosion (Canadian Encyclopedia).

**TABLE 3:** Association between geographical sites and bacteria at phylum rank^*a*^

	Phylum	IndVa lndex	*P*-value	Specificity (A)	Sensitivity (B)
Ekaluktutiak	Actinobacteria	0.70	0.03	0.51	0.95
Akulivik	Fusobacteria	0.91	0.001	0.90	0.92
	Firmicutes	0.81	0.006	0.66	1.00
Inukjuak	Gemmatimonadetes	0.75	0.001	0.59	0.96
Kangiqsualujjuaq	Rhodothermaeota	0.86	0.001	0.93	0.80
	Deinococcus Thermus	0.76	0.001	0.67	0.87
	Ignavibacteriae	0.71	0.001	0.76	0.67
	Planctomycetes	0.69	0.001	0.50	0.930
	Chloroflexi	0.67	0.001	0.62	0.73
	Nitrospirae	0.60	0.005	0.68	0.53
	Thaumarchaeota	0.53	0.003	0.84	0.33
	Calditrichaeota	0.52	0.001	1.00	0.27
Number of permutations: 9999					

^*a*^ Results of the indicator value index, its *P*-value, and its two components which show the specificity and sensitivity.

### Conclusion

The bacterial composition of active gill microbiota in Arctic char differed between groups and was mainly influenced by latitude and, to a lesser extent, air temperature and water type. Dysbiosis patterns were detected in Salluit and Inukjuak, characterized by poor network connectivity and the prevalence of opportunistic pathogens. We hypothesized that such a gill bacterial microbiota dysbiosis was associated with local habitat degradation documented in both communities. Gill microbiota has shown to be a good indicator for monitoring the effect of environmental stresses on fish health (52, 147, 149–151), and more studies are needed to identify how environmental stress impacts Arctic char gill microbiota in the North. A monitoring tool will contribute to the collaborative effort assessing the extent to which current and future threats could impact the fitness of local Arctic char populations.

## MATERIALS AND METHODS

### Fish sampling

Anadromous Arctic char have been sampled from five communities across the Canadian Arctic in freshwaters and saltwaters (Fig. 1; Table 1). In Nunavut, during the ice-free season, four lakes upstream of the Freshwater Creek (Greiner system) and the marine bay facing the community of Ekaluktutiak (Cambridge Bay) on Victoria Island were sampled during August 2018, 2019, and 2020. The four lakes sampled were Greiner Lake (69.18N, −104.99W; 36.9 km^2^), First Lake (69.20N, −104.76W; 3.16 km^2^), Second Lake (69.18N, −104.68W; 268 km^2^), and CBL5 (named Inuhuktok; 69.25N, −104.71; 1.11 km^2^) (152). The fishing sites were selected based on the traditional ecological knowledge shared by the Inuit field guides from the Hunters and Trappers Organization, and adult fish were harvested using gill nets. Fish were killed according to an animal use protocol elaborated by the GLLFAS/WSTD animal care committee, and dissections were made at the Canadian High Arctic Research Station Campus (CHARS). Dissections were carried out in the field under the most sterile conditions possible. Once collected from the net with gloves, the fish were separated and placed in a cool box before being dissected at CHARS with tools cleaned with 70% alcohol and flames between each individual. One- or two-gill arches were taken randomly, without targeting the right or left side of the individual, as the sample size avoided potential bias, and the inter-individual variation between the two sides would not be significant (153). In total, 25 gills from Greiner Lake, 12 from First Lake, 12 from Second Lake, 7 from CBL5, and 7 from Cambridge Bay were used for microbial analysis. The Northern Aquatic Resources lab at the Institute of Systems and Integrative Biology (IBIS, University Laval, Québec, QC, Canada) and the Ministry of Forests, Wildlife, and Parks (Québec, QC, Canada) did sampling across Nunavik. As for Ekaluktutiak in Nunavut, samples were harvested with the Inuit wildlife managers with gillnets or counting weirs (106). In Hudson Bay, samples were collected near the Inukjuak community with 25 individuals from Five Mile Inlet (58.56N, −78.21W). Then, fish were collected near the Akulivik community with seven gills from Korak River (60.75N, −77.63W), three from Chukotat River (60.79N, −78.02W), and three from Saparuajjuit River (60.77,–77.81). In Hudson Strait, 24 individuals were collected in Duquet Lake (62.06N, −74.53W) in the Salluit community. Finally, in Kangiqsualujjuaq (Ungava bay), 10 individuals from George River (58.69N, −65.95W) and 5 from Koroc River (58.89N, −65.79W) were fished. Overall, 63, 25, 13, 24, and 15 Arctic char were caught in Ekaluktutiak, Inukjuak, Akulivik, Salluit, and Kangiqsualujjuaq, respectively (Fig. 1). Thus, a total of 140 fish belonging to the five regions could be dissected for the analysis of Arctic char gill microbiota. One- or two-gill arches were collected for each individual and preserved in Nucleic Acid Preservation buffer (154, 155).

Moreover, weight and fork length were measured for each fish except for some coming from George River (Kangiqsualujjuaq), Korak, and Chukotat River (Akulivik). Fulton index, which indicates the physiological condition of the fishes, was also calculated according to Froese (156): *K* = 100 × (*W*/*L*)^3^, with weight (*W*) in grams and length (*L*) in centimeters. Normality was assessed with Shapiro’s test, and homoscedasticity was assessed with Levene’s test (157), to compare morphological traits between communities. Those conditions were not respected. The Kruskal-Wallis test was therefore performed in R version 3.4.2 (158) followed by multiple pairwise comparisons between groups, i.e., function “wilcoxtest()” with the argument “p.adjust.method = BH” to adjust *P*-values for multiple comparisons using Benjamini and Hochberg’s false discovery rate (FDR).

### 16S rRNA gene library construction

Gills preserved in NAP buffer were washed with PBS 1×, pH 7.4. Then, 100 mg of gill tissues per individual was used to extract RNA using Trizol reagent (cat #15596026, Thermo Fisher Scientific). Throughout the libraries’ construction, 12 controls were processed with the same protocol as the samples. These controls were negative controls that followed the same protocols (RNA extraction, reverse transcription, PCR, and DNA purification) as the rest of the samples to ensure no contamination of the reagents throughout the laboratory. Once RNA was extracted, reverse transcription PCR was done using the qScript cDNA Synthesis Kit (cat #95048-100) from QuantaBio (Beverly, MA, USA). Finally, the V4 fragment of the universal microbial marker rRNA 16S gene was amplified with a first PCR using the primers 519-F (5′-ACA CTC TTT CCC TAC ACG ACG CTC TTC CGA TCT CAG CMG CCG CGG TAA -3), 745-R (5′- GTG ACT GGA GTT CAG ACG TGT GCT CTT CCG ATC TGA CTA CHV GGG TAT CTA ATCC -3′) (Sigma-Aldrich, St. Louis, MO, USA) and using the enzyme Q5 High Fidelity DNA Polymerase with the manufacturer’s standard protocol (New England, Biolabs). Initial denaturation was at 98°C for 2 minutes, denaturation was at 98°C for 10 seconds, then annealing was at 60°C for 30 seconds, followed by elongation at 72°C for 30 seconds, and the final elongation at 72°C for 10 minutes. After 35 cycles, electrophoresis on 2% agarose gels was done to verify the successful amplification of the V4 region. Samples were then purified with AMPure beads [cat #A63880, Beckman Coulter, Pasadena (CA), USA] and quantified by Nanodrop. A second PCR was performed to barcode samples with two indexes. For this PCR, the final elongation was at 72°C for 10 minutes, and 12 cycles were programmed. As for the first PCR, 2% agarose gels were used, followed by purification. Finally, barcoded samples were pooled, and the smallest concentration of DNA was used to equilibrate the quantity of DNA for each sample in the pool. The sequencing was made on Illumina MiSeq in paired-end mode (2 × 300 bp) at the IBIS Genomics Platform (Université Laval, Québec, QC, Canada).

### Bioinformatics

After sequencing, quality filtering and trimming were done to remove reads with poor quality with dada2 (159) using R v 3.4.2 (158). According to visualization of the Phred scores, truncations were made at 275 for the forward reads and 270 for reverse reads (truncLen). The NAs (not available) data were eliminated, and the threshold of expected error was 4 for the forward reads and 5 for the reverse reads (maxEE). Then, the sequences were cut at the beginning of 5 pb to have better quality sequences (trimLen = 5), and a prediction model was used to correct on the reads to avoid data loss of those reads (160). Finally, ASVs were clustered with dada2 with an identity threshold of 97%. Chimeras were removed, and ASVs were decontaminated with control reads. The NCBI 16S Microbial Database was used to assign taxonomy to ASVs with dada2 (161). ASV raw counts, metadata, and taxonomy tables were imported into the R package phyloseq (162). From this phyloseq object, ASVs with a mean relative activity <1e−5 and samples with a total count <10,000 were filtrated. After normalization steps, 8,531 ASV remained, and 4 samples were discarded because of their low total count: one from Cambridge Bay, one from Five Mile Inlet, and two from Duquet Lake.

### Environmental data

Water sampling and data measurement in Ekaluktutiak (Victoria Island, Nunavut) were performed by the Laboratory of Aquatic Sciences of the Université du Quebec à Chicoutimi (QC, Canada). Water temperature, conductivity, O_2_ concentration, O_2_ saturation, and salinity were measured with a Ruskin RBR Concerto probe (Ottawa, ON, Canada). Environment and Climate Change Canada at the National Laboratory for Environmental Testing (Burlington, ON, Canada) analyzed DOC content in the water samples [see section “Materials and Methods” in references (152, 163)]. The environmental data in Nunavik were estimated using ArcGIS software v10.4 (164), BIO-Oracle v2.0 (165), Marspec (166), and WorldClim v2.0 (167) [see section “2. Methods” in reference (168)]. Air temperatures were collected for Ekaluktutiak, Salluit, Akulivik, Inukjuak, and Kangiqsualujjuaq using the Environment and Climate Change Canada database (climate.weather.gc.ca) (Table S1). From these data, we calculated the average air temperature in the month in which each group of fish was caught. As with the morphometric data, grouping all the environmental information for all the sites was challenging. In Ekaluktutiak, we do not have environmental data from 2020 (10 samples) and from Cambridge Bay because the RBR probe was not available that day. Therefore, another data set with a different number of samples was used for the rRNA 16s analysis with environmental data (Fig. 6). Table S2 makes it easier for the reader to understand the different data sets used for the different analyses.

### Statistics

#### Relative activity

Barplots with mean and standard error, representing the relative activity of the most active ASVs (Fig. 2; Fig. S7), were made using the package ggplot2 (169) on RStudio (170). Non-parametric Kruskal-Wallis and Wilcoxon tests assessed the significant differences with a *P*-value adjusted with Benjamini-Hochberg’s FDR correction (*P* < 0.05). An ASV activity heatmap (Fig. 3) was performed with METAGENassist (171). During filtration, 307 out of 16,801 variables with less than 50% zeros and passing the interquartile range filter were conserved to construct the heatmap. Following a Pareto scaling normalization, the heatmap was built with Pearson distances using Ward’s clustering algorithm (171).

#### Alpha diversity

The alpha diversity index Pielou, an index of evenness (172), and Chao1, an estimator of species richness unbiased by low activity taxa (173), were calculated and represented in Fig. 4A and B. Phylogenetic trees were constructed with the neighbor-joining method (174) to calculate Faith’s phylogenetic diversity metric, an alpha diversity index based on phylogenetic distances. This was made using the package btools in R (175) (Fig. 4C). When normality (Shapiro-Wilk test) and homoscedasticity (Levene test) were not met, the non-parametric test Kruskal-Wallis was performed. To assess the significant differences in alpha diversity between communities or specific sites, multiple pairwise comparisons between groups (Wilco text) with Benjamini-Hochberg correction were used. Otherwise, an ANOVA followed by a Tukey test was used.

#### Beta diversity

To compare the microbiota composition between sites, a principal coordinates analysis with weighted UniFrac distances (Fig. 5; 175) was performed to visualize the dissimilarities between the different geographical groups using the ggplot2 package (169). A permutation-based multivariate analysis of variance [Table 2, (176)] was performed to assess the differences in microbiota composition between geographical groups using the adonis function in the vegan package in RStudio (176). A Benjamini-Hochberg correction was made to account for the unbalanced experimental design and the susceptibility of PERMANOVA to the heterogeneous dispersion of factor groups (177). Finally, an analysis of multivariate homogeneity of group dispersion (variances) was done with the betadisper function in the vegan package. Another PCoA was processed by grouping “Freshwater” and “Saltwater” to assess the impact of water types on analyses, instead of communities. A nonmetric multidimensional scaling (Fig. 6; 147) on weighted UniFrac distances was fitted using the dplyr package (178) in RStudio. For this analysis, we removed the samples without any environmental data. A new metadata table and a new phyloseq object were created with the 120 remaining samples. Then, the envfit function from the vegan package was used to fit the environmental parameters on the NMDS plot with 9,999 permutations. This function calculates multiple regressions of the different environmental variables in the NMDS ordination. Because of the number of tested variables, the Bonferroni correction was performed for the *P*-value with the function p.adjust. A plot was made using ggplot2 to visualize the NMDS axis with the weighted UniFrac distances of each sample and the environmental parameters fitted. Spearman’s correlations between all environmental variables (salinity, water temperature, air temperature, chlorophyll-a, and O_2_ concentration) were calculated to take independent variables in our analysis.

#### Indicator species analysis

To associate the bacteria at a different taxonomic rank (phylum, genus, and species) with various geographical sites, the function “multipatt” from the Indicspecies package was performed. It allowed us to have indicator species for each community with the indicator value index (179, 180). For each indicator value, a specificity index (the probability that the sites where we found an indicator species fit with the target sites of this indicator species) and a sensitivity index (the probability of finding the indicator species in the target sites associated with this species) were associated. A permutational test is performed in this function to assess the statistical significance for each association with an IndVal index >0.5 and *P* < 0.05 (Table 3).

#### Co-activity networks

Spearman’s correlation coefficient was calculated between each pair of ASV at the genus level in Rstudio (v 4.0.5) with the function “rcorr” from the Hmisc package (181) for each geographical site. A threshold of −0.4 and 0.4 for the Spearman’s coefficient was set, and *P*-values were adjusted with the false discovery rate method with p-FDR <0.05 (182). Then, the resulting five tables combining activities and correlations for the top 50 most active taxa at the genus rank were visualized using Cytoscape (v 3.5.1) (183), resulting in five co-activity networks for the five different geographical sites (Fig. 7). Nodes represent the different active taxa at a genus level, their colors represent the phylum, and their size shows the 16S rRNA gene expression level as a proxy for overall transcriptomic activity. The edges between nodes represent the correlation between taxa. The green edges show positive correlations, while the red edges show negative correlations. Finally, three metrics have been extracted from the networks with the function “Network Analyzer” in Cytoscape: degree (DG), neighborhood connectivity (NC), and closeness centrality (CC). Those network topological parameters allow a better understanding of the dynamics in different networks. DG is the number of edges connected from one node to another (184). The more a node has edges, the more it is locally connected, indicating its relevance in the network (185). CC is a qualitative measure that indicates if a node is close to the other nodes in the network (186). The shortest path length to spread information from one node to another is represented, and it indicates if the node has an important influence in the network and how it can interact with other nodes (184). Finally, NC is a quantitative measure that calculates the average connectivity of a node to the other nodes in the network. It indicates how the node impacts in the network dynamics (187).

## Data Availability

Raw sequence reads from this study were deposited on the NCBI Sequence Read Archive under the following BioProject number: PRJNA944667.

## SUPPLEMENTAL MATERIAL

Figure S1 spectrum.02943-23-s0001.docx Boxplot of the distances to the centroid.

Figure S2 spectrum.02943-23-s0002.docx NMDS with water temperature and pH. fitted

Figure S3 spectrum.02943-23-s0003.docx Boxplot methodological bias.

Figure S4 spectrum.02943-23-s0004.docx PCoA latitude.

Figure S5 spectrum.02943-23-s0005.docx Beta diversity - PCoA and types of water.

Figure S6 spectrum.02943-23-s0006.docx Violin plot of three different topological metrics: Degree (DG), Connectivity Centrality (CC), and Neighborhood Closeness (NC) to describe the connectivity in each community.

Figure S7 spectrum.02943-23-s0007.docx Relative activity of the 180 most active ASVs at genus rank (A) and of the 150 most active ASVs at species rank (B).

Table S1 spectrum.02943-23-s0008.docx Physico-chemical data from sampling sites.

Table S2 spectrum.02943-23-s0009.docx Number of samples for the different sampling sites according to the different datasets for the various analyses and figures.

Table S3 spectrum.02943-23-s0010.docx Topological metrics.

ASM does not own the copyrights to Supplemental Material that may be linked to, or accessed through, an article. The authors have granted ASM a non-exclusive, world-wide license to publish the Supplemental Material files. Please contact the corresponding author directly for reuse.
