# Supplementary material for: Characterization of gill bacterial microbiota in wild Arctic char (Salvelinus alpinus) across lakes, rivers, and bays in the Canadian Arctic ecosystems
Source: Microbiol Spectr. 2024 Feb 8;12(3):e02943-23. doi: 10.1128/spectrum.02943-23 (PMC10923216; doi:10.1128/spectrum.02943-23)
Supplement: Figure S1 — Boxplot of the distances to the centroid. [file spectrum.02943-23-s0001.docx]

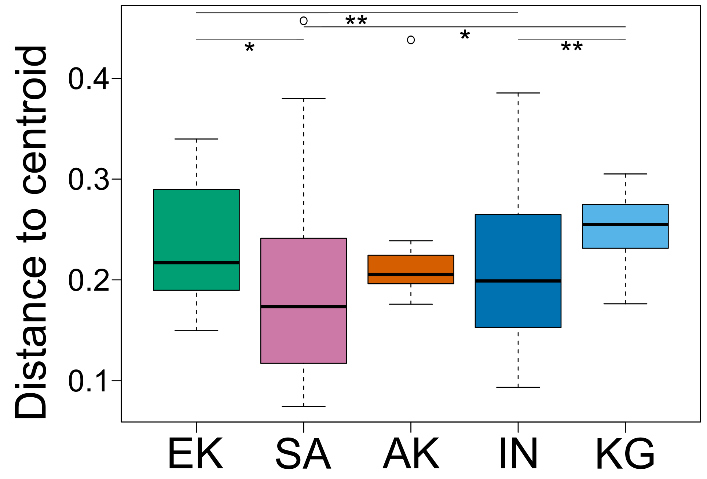


**Figure S1**: Boxplot of the distances to the centroid of the UniFrac weighted distances in each geographical group; Ekaluktutiak (EK), Salluit (SA), Akulilvik (AK), Inukjuak (IN), Kangiqsualujjuaq (KG), from the multivariate homogeneity of groups dispersions (variances) analysis. Statistical significances: ‘***’ < 0.001, ‘**’ < 0.01, ‘*’ < 0.05.
