# Supplementary material for: Characterization of gill bacterial microbiota in wild Arctic char (Salvelinus alpinus) across lakes, rivers, and bays in the Canadian Arctic ecosystems
Source: Microbiol Spectr. 2024 Feb 8;12(3):e02943-23. doi: 10.1128/spectrum.02943-23 (PMC10923216; doi:10.1128/spectrum.02943-23)
Supplement: Figure S2 — NMDS with water temperature and pH. fitted [file spectrum.02943-23-s0002.docx]

**
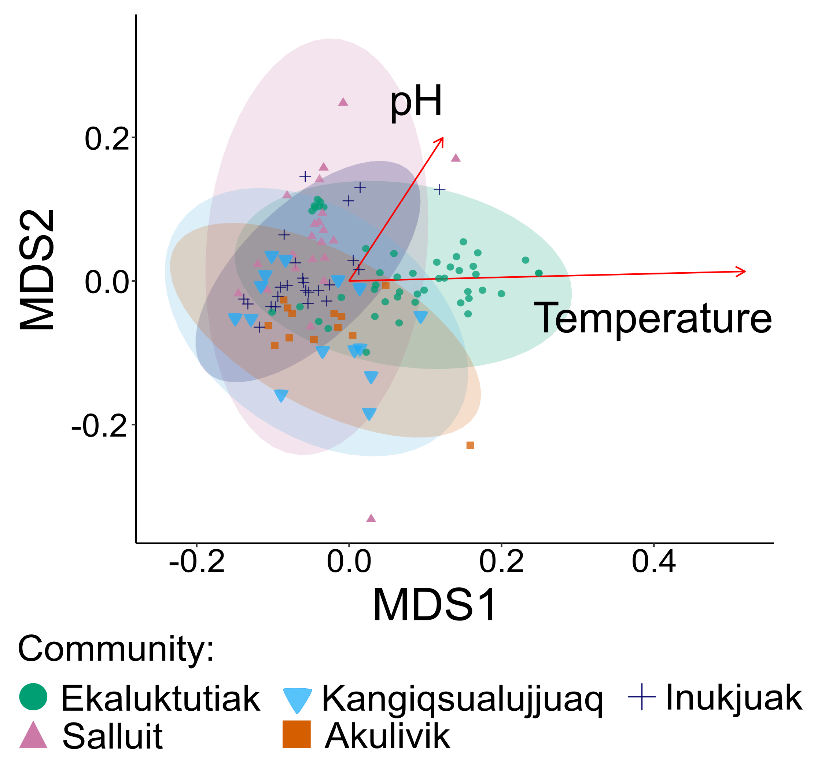
**

**Figure S2:** Beta diversity. NMDS on weighted UniFrac distances with environmental independent parameters fitted. The environmental parameters were represented across the samples from the five different communities in the Arctic: Ekaluktutiak (green), Salluit (pink), Akulivik (orange), Inukjuak (blue), and Kangiqsualujjuaq (turquoise)*.* Water temperature was significantly correlated to the bacterial relative activity in Arctic char gill microbiota.
