# Supplementary material for: Characterization of gill bacterial microbiota in wild Arctic char (Salvelinus alpinus) across lakes, rivers, and bays in the Canadian Arctic ecosystems
Source: Microbiol Spectr. 2024 Feb 8;12(3):e02943-23. doi: 10.1128/spectrum.02943-23 (PMC10923216; doi:10.1128/spectrum.02943-23)
Supplement: Figure S3 — Boxplot methodological bias. [file spectrum.02943-23-s0003.docx]

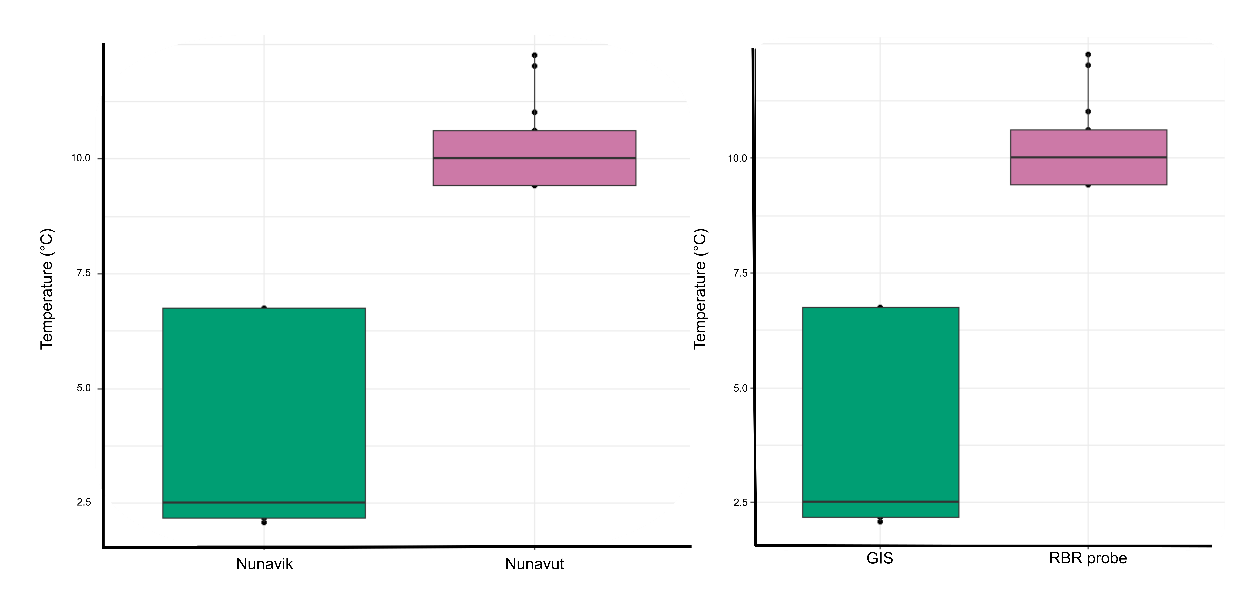


**Figure S3:** Boxplots of temperature as a function of region (Nunavut/Nunavik) and temperature measurement method (GIS and RBR probes).
