# Supplementary material for: Characterization of gill bacterial microbiota in wild Arctic char (Salvelinus alpinus) across lakes, rivers, and bays in the Canadian Arctic ecosystems
Source: Microbiol Spectr. 2024 Feb 8;12(3):e02943-23. doi: 10.1128/spectrum.02943-23 (PMC10923216; doi:10.1128/spectrum.02943-23)
Supplement: Figure S4 — PCoA latitude. [file spectrum.02943-23-s0004.docx]

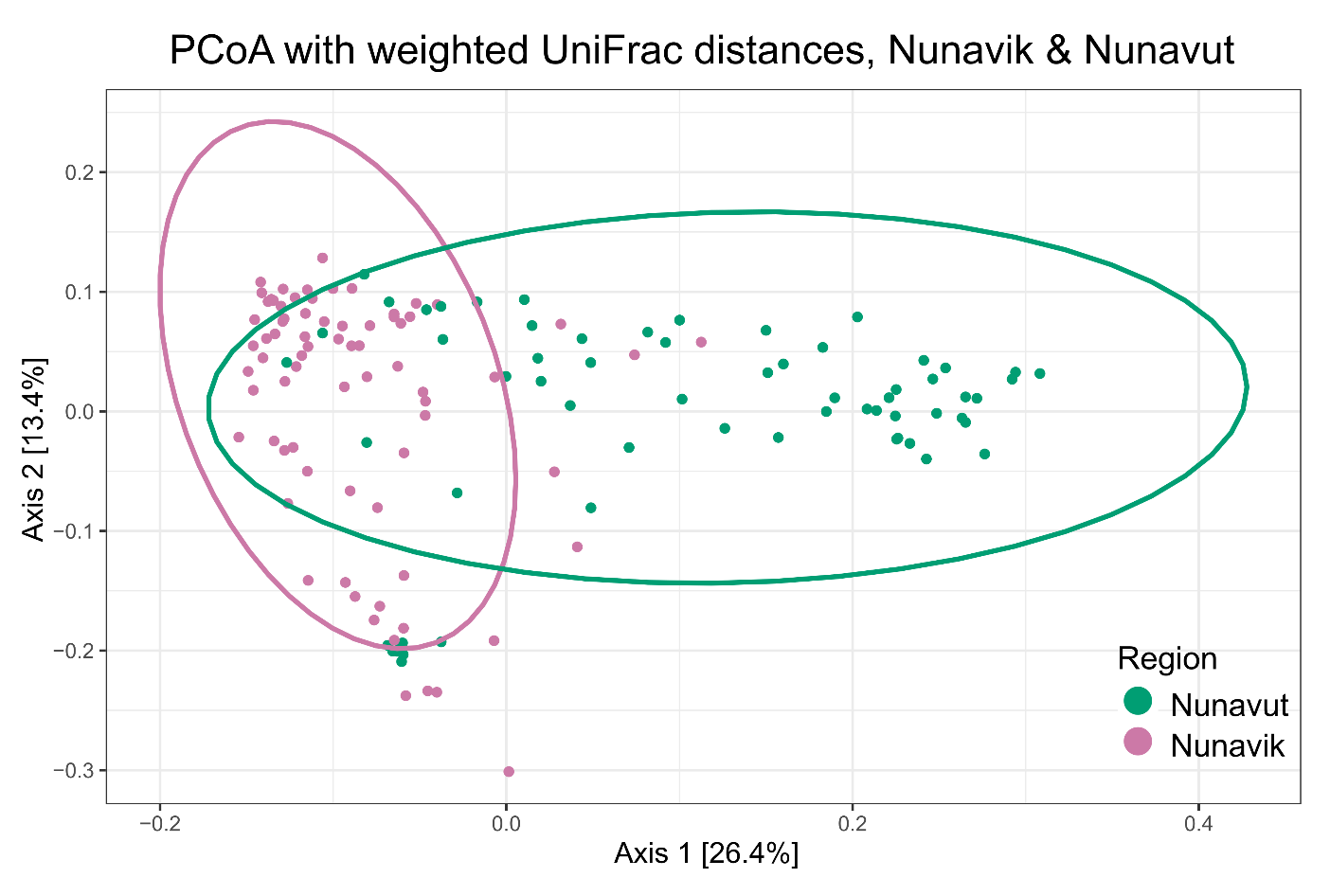


**Figure S4**: Beta diversity. Principal Coordinates Analysis (PcoA) of the samples from the five different communities in the Nunavik in pink and the Nunavut in green. The weighted UniFrac distances were used to construct PCoA, and a multivariate analysis of variance with 9999 permutations was performed to obtain the p-value.
