# Supplementary material for: Characterization of gill bacterial microbiota in wild Arctic char (Salvelinus alpinus) across lakes, rivers, and bays in the Canadian Arctic ecosystems
Source: Microbiol Spectr. 2024 Feb 8;12(3):e02943-23. doi: 10.1128/spectrum.02943-23 (PMC10923216; doi:10.1128/spectrum.02943-23)
Supplement: Figure S5 — Beta diversity - PCoA and types of water. [file spectrum.02943-23-s0005.docx]

**
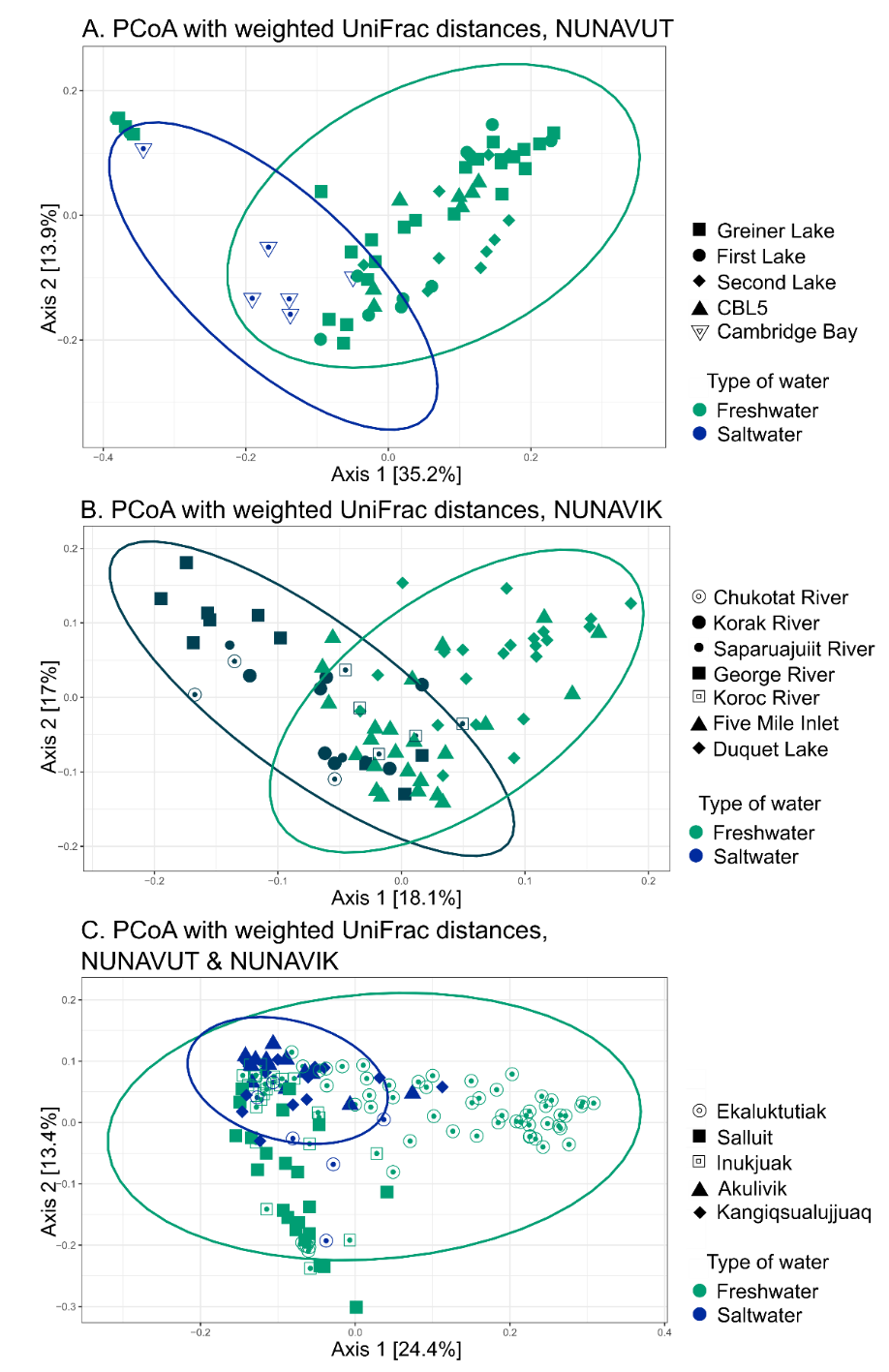
**

**Figure S5**: Beta diversity. Principal Coordinates Analysis (PcoA) of the samples from the five different communities in the Arctic: Ekaluktutiak, Salluit, Akulivik, Inukjuak, and Kangiqsualujjuaq. Dots from freshwater sites were green, and dots from saltwater sites were blue. The weighted UniFrac distances were used to construct PCoA, and a multivariate analysis of variance with 9999 permutations was performed to obtain the p-value.
