# Supplementary material for: Characterization of gill bacterial microbiota in wild Arctic char (Salvelinus alpinus) across lakes, rivers, and bays in the Canadian Arctic ecosystems
Source: Microbiol Spectr. 2024 Feb 8;12(3):e02943-23. doi: 10.1128/spectrum.02943-23 (PMC10923216; doi:10.1128/spectrum.02943-23)
Supplement: Figure S6 — Violin plot of three different topological metrics: Degree (DG), Connectivity Centrality (CC), and Neighborhood Closeness (NC) to describe the connectivity in each community. [file spectrum.02943-23-s0006.docx]

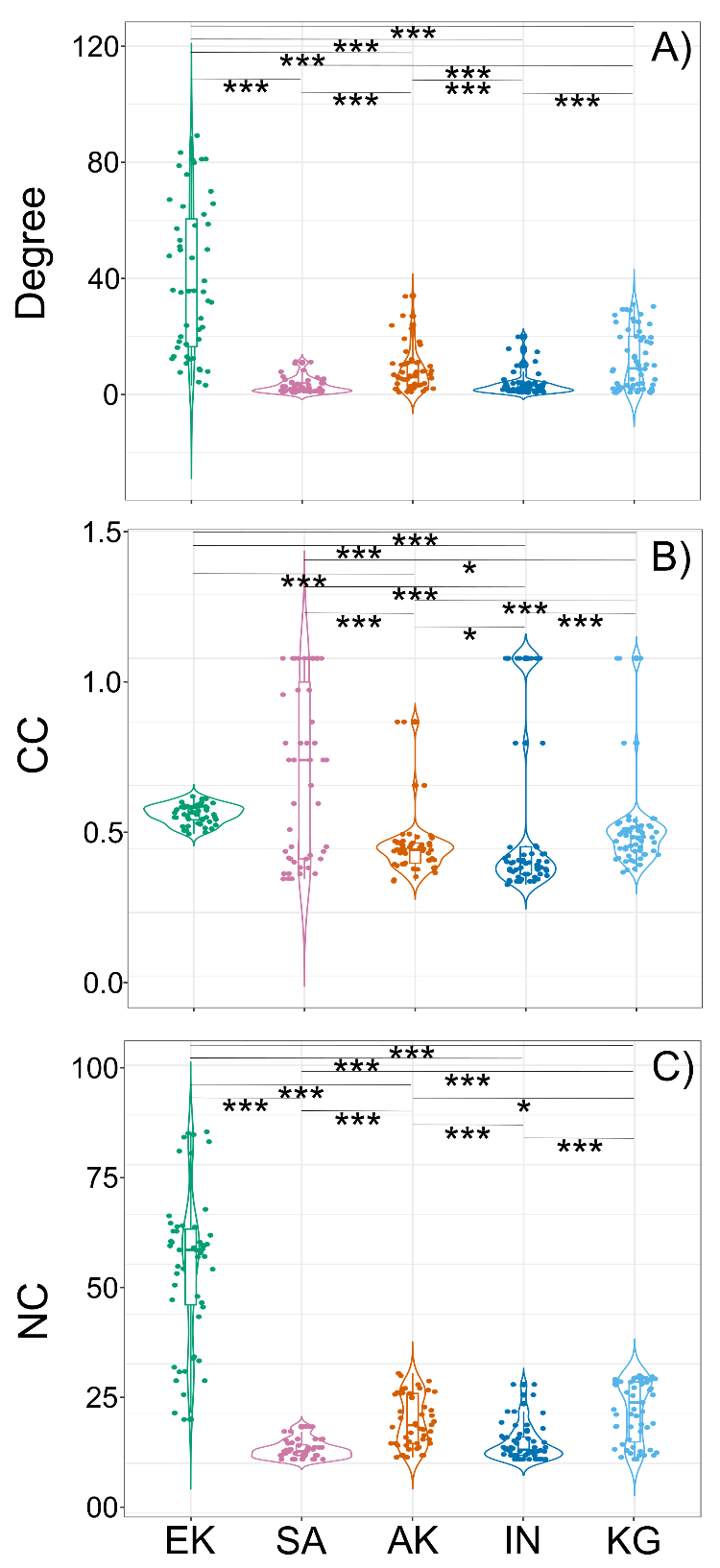


**Figure S6**: Violin plot of three different topological metrics: Degree (DG), Connectivity Centrality (CC), and Neighborhood Closeness (NC) to describe the connectivity in each community. Statistical significances: ‘***’ < 0.001, ‘**’ < 0.01, ‘*’ < 0.05
