# Supplementary material for: Characterization of gill bacterial microbiota in wild Arctic char (Salvelinus alpinus) across lakes, rivers, and bays in the Canadian Arctic ecosystems
Source: Microbiol Spectr. 2024 Feb 8;12(3):e02943-23. doi: 10.1128/spectrum.02943-23 (PMC10923216; doi:10.1128/spectrum.02943-23)
Supplement: Figure S7 — Relative activity of the 180 most active ASVs at genus rank (A) and of the 150 most active ASVs at species rank (B). [file spectrum.02943-23-s0007.docx]

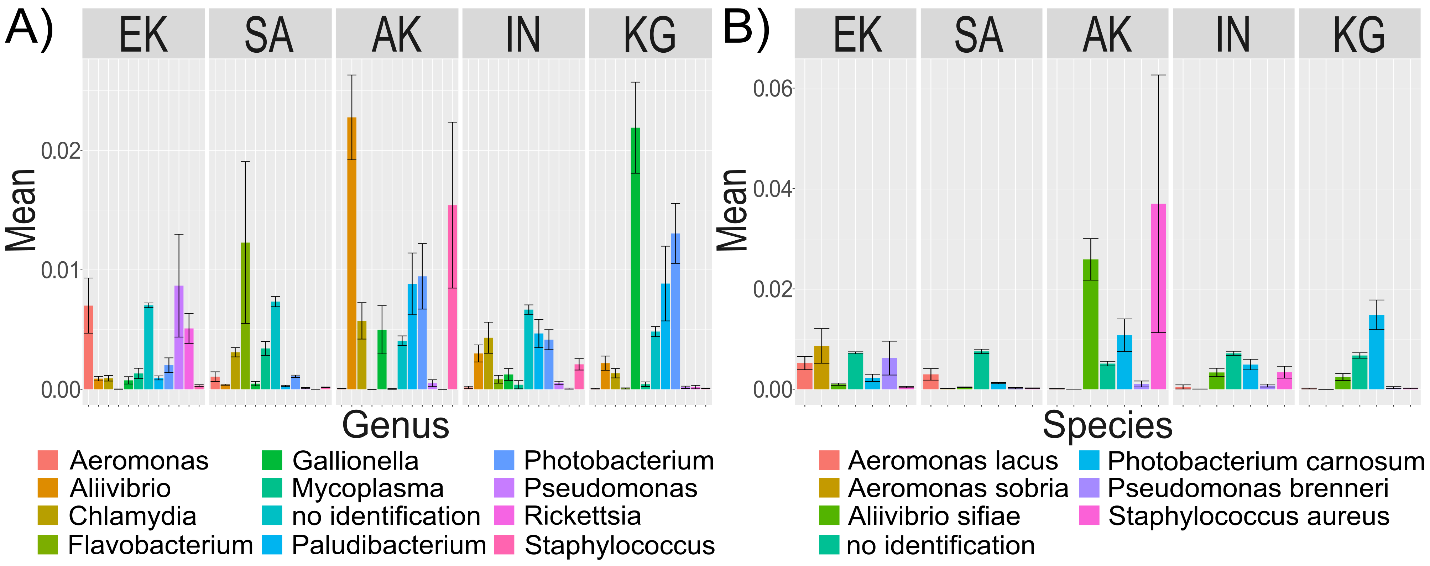


**Figure S7**: Relative activity of the 180 most active ASVs at genus rank (A.) and of the 150 most active ASVs at species rank (B.) found in the microbiota of the Arctic chars’ gills across the five different communities Ekaluktutiak (EK), Salluit (SA), Akulivik (AK), Inukjuak (IN) and Kangiqsualujjuaq(K
