# Supplementary material for: Characterization of gill bacterial microbiota in wild Arctic char (Salvelinus alpinus) across lakes, rivers, and bays in the Canadian Arctic ecosystems
Source: Microbiol Spectr. 2024 Feb 8;12(3):e02943-23. doi: 10.1128/spectrum.02943-23 (PMC10923216; doi:10.1128/spectrum.02943-23)
Supplement: Table S1 — Physico-chemical data from sampling sites. [file spectrum.02943-23-s0008.docx]

**Table S1:** Physico-chemical data from sampling sites. Mean of water temperature (°C), oxygen concentration (mg/L), salinity (PSU), DOC (mg/L), pH, and chlorophyll-a concentration (ug/L) at various sampling sites in Ekaluktutiak, Salluit, Akulivik, Inukjuak, and Kangiqsualujjuaq.

| Community | Location | Water Temperature (°C) | Air Temperature (°C) | [O2] (mg/L) | Salinity (PSU) | pH | DOC (mg/L) | chlA (ug/L) |
| --- | --- | --- | --- | --- | --- | --- | --- | --- |
| Ekaluktutiak | Greiner Lake | 9.43 | 4.95 | 18.05 | 0.13 | 8.1 | 6.1 | 2.34 |
|  |  | 12.25 | 8.11 | 11.42 | 0.11 | 7.99 | 3.7 | 1.88 |
|  |  | NA | 8.21 | NA | 0.17 | NA | 3.71 | NA |
|  | First Lake | 9.95 | 4.95 | 12.45 | 0.12 | 8.7 | 4.2 | 2.04 |
|  |  | 12.02 | 8.11 | 11.2 | 0.11 | 7.99 | 3.8 | 2.12 |
|  |  | NA | 8.21 | NA | 0.15 | NA | NA | NA |
|  | Second Lake | 10.01 | 4.95 | 12.96 | 0.12 | 8.6 | 4.3 | 1.60 |
|  |  | 11.02 | 8.11 | 11.4 | 0.10 | 7.88 | 4.1 | 2.44 |
|  | CBL5  (Inuhuktok) | 10.61 | 8.11 | 11.69 | 0.13 | 8.13 | 3.6 | 1.82 |
|  | Cambridge Bay | NA | 4.95 | NA | NA | NA | NA | NA |
|  |  | NA | 8.11 | NA | NA | NA | NA | NA |
| Salluit | Duquet Lake | 2.17 | -2.35 | NA | NA | 8.20 | NA | 0.29 |
| Akulivik | Chukotat River | 5.81 | 5.21 | NA | NA | 8.20 | NA | 0.34 |
|  | Saparuajuiit River | 4.16 | 5.21 | NA | NA | 8.20 | NA | 0.45 |
|  | Korak River | 5.72 | 5.21 | NA | NA | 8.20 | NA | 0.36 |
| Inukjuak | Five Mile Inlet | 6.75 | 7.21 | NA | NA | 8.20 | NA | 0.30 |
| Kangiqsualujjuaq | George River | 2.52 | 11.59 | NA | NA | 8.186 | NA | 0.57 |
|  | roc River | 2.08 | 18.86 | NA | NA | 8.19 | NA | 0.55 |
