# Supplementary material for: Characterization of gill bacterial microbiota in wild Arctic char (Salvelinus alpinus) across lakes, rivers, and bays in the Canadian Arctic ecosystems
Source: Microbiol Spectr. 2024 Feb 8;12(3):e02943-23. doi: 10.1128/spectrum.02943-23 (PMC10923216; doi:10.1128/spectrum.02943-23)
Supplement: Table S2 — Number of samples for the different sampling sites according to the different datasets for the various analyses and figures. [file spectrum.02943-23-s0009.docx]

**Table S2:**  Number of samples for the different sampling sites according to the different datasets for the various analyses and figures.

| **Local communities** | **Sampling sites** | **Total fish caught (Fig. 1)** | **Morphometric data (Table S1)** | **Bioinformatics DNA filtering (Fig. 2, 3, 4, 5, 7)** | **Environ-mental data (Fig. 6)** |
| --- | --- | --- | --- | --- | --- |
| **Ekaluktutiak (EK)** | Greiner Lake | 25 | 25 | 25 | 20 |
|  | First Lake | 12 | 12 | 12 | 7 |
|  | Second Lake | 12 | 12 | 12 | 12 |
|  | CBL5 | 7 | 7 | 7 | 7 |
|  | Cambridge Bay | 7 | 7 | 6 | NA |
|  | **Total EK** | **63** | **63** | **62** | **46** |
| **Akulivik (AK)** | Saparuajuiit River | 3 | 3 | 3 | 3 |
|  | Chukotat River | 3 | 2 | 3 | 3 |
|  | Korak River | 7 | 4 | 7 | 7 |
|  | **Total AK** | **13** | **9** | **13** | **13** |
| **Inukjuak (IN)** | Five Mile Inlet | 25 | 25 | 24 | 24 |
|  | **Total IN** | **25** | **25** | **24** | **24** |
| **Kangiqsualujjuaq (KG)** | George River | 10 | 0 | 10 | 10 |
|  | Koroc River | 5 | 5 | 5 | 5 |
|  | **Total KG** | **15** | **5** | **15** | **15** |
| **Salluit (SA)** | Duquet Lake | 24 | 24 | 22 | 22 |
|  | **Total SA** | **24** | **24** | **22** | **22** |
| **Total** | **All sites** | **140** | **126** | **136** | **120** |
