# Supplementary material for: Characterization of gill bacterial microbiota in wild Arctic char (Salvelinus alpinus) across lakes, rivers, and bays in the Canadian Arctic ecosystems
Source: Microbiol Spectr. 2024 Feb 8;12(3):e02943-23. doi: 10.1128/spectrum.02943-23 (PMC10923216; doi:10.1128/spectrum.02943-23)
Supplement: Table S3 — Topological metrics. [file spectrum.02943-23-s0010.docx]

**Table S3**: Topological metrics (Degree (DG), Connectivity Centrality (CC), and Neighborhood Closeness (NC)) for the most abundant genus (A) and for the genera that present negative correlations in the interaction network with the 50 most active genera in Ekaluktutiak (B), Akulivik (C), Kangiqsualujjuaq (D), and Inukjuak (E). Red: degree less than the mean degree for all the nodes, Orange: degree close to the mean, and green: degree higher than the mean.

| **A)** | **Total Positive**  **Correlations** | **Total Negative**  **Correlations** | **Most abundant genus** | **Phylum** | **Abundance** | **DG** | **CC** | **NC** |
| --- | --- | --- | --- | --- | --- | --- | --- | --- |
| **Ekaluktutiak** | 7975 | 58 | *Photobacterium* | **Proteobacteria** | 53714 | 32 | 0,41 | 49,47 |
|  |  |  | *Flavobacterium* | **Bacteroidetes** | 67559 | 13 | 0,35 | 58,31 |
|  |  |  | *Aeromonas* | **Proteobacteria** | 77658 | 51 | 0,39 | 55,78 |
|  |  |  | *Rickettsia* | **Proteobacteria** | 88166 | 39 | 0,42 | 59,72 |
|  |  |  | *Pseudomonas* | **Proteobacteria** | 118617 | 36 | 0,37 | 51,97 |
| **Salluit** | 288 | 0 | *Lactobacillus* | **Firmicutes** | 7048 | 4 | 0,15 | 4 |
|  |  |  | *Photobacterium* | **Proteobacteria** | 7324 | 2 | 1 | 1 |
|  |  |  | *Mycoplasma* | **Tenericutes** | 12247 | 1 | 0,67 | 2 |
| **Akulivik** | 930 | 17 | *Moritella* | **Proteobacteria** | 9200 | 3 | 0,19 | 5 |
|  |  |  | *Flavobacterium* | **Bacteroidetes** | 19330 | 1 | 0,12 | 2 |
|  |  |  | *Gallionella* | **Proteobacteria** | 22016 | 1 | 0,13 | 2 |
| **Inukjuak** | 824 | 2 | *Chlamydia* | **Chlamydiae** | 19235 | 1 | 0,16 | 3 |
|  |  |  | *Pseudomonas* | **Proteobacteria** | 20563 | 4 | 0,12 | 3,25 |
|  |  |  | *Aliivibrio* | **Proteobacteria** | 38682 | 1 | 0,67 | 2 |
| **Kangiqsualujjuaq** | 952 | 43 | *Flavobacterium* | **Bacteroidetes** | 17383 | 1 | 0,24 | 20 |
|  |  |  | *Aliivibrio* | **Proteobacteria** | 17841 | 11 | 0,29 | 15,27 |
|  |  |  | *Photobacterium* | **Proteobacteria** | 112256 | 1 | 0,21 | 8 |

| **B) Network for the 50 taxa the most abundant** | | | | **Correlations** | | | **Topological metrics** | | | | |
| --- | --- | --- | --- | --- | --- | --- | --- | --- | --- | --- | --- |
| **Genus (Ekaluktutiak)** | | **Phylum** | **Abundance** | **Positive** | **Negative** | | **DG** | | **NC** | **CC** | |
| *Deefgea* | | **Proteobacteria** | 3756 | 22 | **1** | | 66 | | 55,5 | 0,43 | |
| *Mycoplasma* | | **Tenericutes** | 13633 | 8 | **1** | | 22 | | 36,82 | 0,37 | |
| *Sphingomonas* | | **Proteobacteria** | 6303 | 5 | **1** | | 16 | | 26,31 | 0,37 | |
| *Tychonema* | | **Cyanobacteria** | 9744 | 21 | **1** | | 76 | | 54,17 | 0,45 | |
| *Photobacterium* | | **Proteobacteria** | 53714 | 11 | **2** | | 32 | | 49,47 | 0,41 | |
| *Pseudoalteromonas* | | **Proteobacteria** | 1932 | 9 | **2** | | 26 | | 62,15 | 0,39 | |
| *Solitalea* | | **Bacteroidetes** | 2677 | 18 | **2** | | 81 | | 55,02 | 0,46 | |
| *Terrimicrobium* | | **Verrucomicrobia** | 5626 | 13 | **2** | | 48 | | 63,79 | 0,42 | |
| *Psychromonas* | | **Proteobacteria** | 3576 | 10 | **5** | | 65 | | 78,43 | 0,45 | |
| *Rickettsia* | | **Proteobacteria** | 88166 | 6 | **5** | | 39 | | 59,72 | 0,42 | |
| **C) Network for the 50 taxa the most abundant** | | | | **Correlations** | | | | **Topological metrics** | | | |
| **Genus (Akulivik)** | **Phylum** | | **Abundance** | **Positive** | | **Negative** | | **DG** | **NC** | | **CC** |
| *Algoriphagus* | **Bacteroidetes** | | 479 | 7 | | **1** | | 17 | 17,53 | | 0,26 |
| *Gaetbulibacter* | **Bacteroidetes** | | 415 | 12 | | **1** | | 34 | 17,32 | | 0,31 |
| *Winogradskyella* | **Bacteroidetes** | | 163 | 3 | | **1** | | 11 | 11,36 | | 0,27 |
| *Yoonia* | **Proteobacteria** | | 144 | 8 | | **1** | | 18 | 17,44 | | 0,28 |
| *Amylibacter* | **Proteobacteria** | | 240 | 2 | | **2** | | 4 | 12,25 | | 0,26 |
| *Crocinitomix* | **Bacteroidetes** | | 537 | 8 | | **2** | | 19 | 17,58 | | 0,30 |
| *Lacinutrix* | **Bacteroidetes** | | 6661 | 4 | | **2** | | 7 | 13,00 | | 0,26 |
| *Polaribacter* | **Bacteroidetes** | | 656 | 9 | | **2** | | 24 | 17,79 | | 0,31 |
| *Acinetobacter* | **Proteobacteria** | | 764 | 0 | | **3** | | 8 | 18,63 | | 0,25 |
| *Hassallia* | **Cyanobacteria** | | 3622 | 2 | | **4** | | 10 | 8,50 | | 0,28 |
| *Planktothrix* | **Cyanobacteria** | | 159 | 1 | | **5** | | 8 | 12,00 | | 0,28 |

| **D) Network for the 50 taxa the most abundant** | | | **Correlations** | | **Topological metrics** | | | |
| --- | --- | --- | --- | --- | --- | --- | --- | --- |
| **Genus (Kangiqsualujjuaq)** | **Phylum** | **Abundance** | **Positive** | **Negative** | **DG** | **NC** | **CC** | |
| *Altererythrobacter* | **Proteobacteria** | 864 | 15 | 1 | 25 | 22,04 | 0,36 | |
| *Boseongicola* | **Proteobacteria** | 1527 | 19 | 1 | 30 | 20,43 | 0,37 | |
| *Flavimaricola* | **Proteobacteria** | 244 | 18 | **1** | 28 | 21,36 | 0,35 | |
| *Gaetbulibacter* | **Bacteroidetes** | 2176 | 15 | 1 | 26 | 21,73 | 0,37 | |
| *Nonlabens* | **Bacteroidetes** | 277 | 9 | 1 | 18 | 20,33 | 0,33 | |
| *Aliivibrio* | **Proteobacteria** | 17841 | 2 | 2 | 11 | 15,27 | 0,29 | |
| *Litorilinea* | **Chloroflexi** | 380 | 13 | 2 | 24 | 21,25 | 0,35 | |
| *Parasphingorhabdus* | **Proteobacteria** | 972 | 18 | **2** | 31 | 20,48 | 0,38 | |
| *Roseovarius* | **Proteobacteria** | 301 | 14 | **2** | 29 | 19,86 | 0,36 | |
| *Sulfurimonas* | **Proteobacteria** | 1520 | 17 | 2 | 29 | 21,41 | 0,34 | |
| *Aquaspirillum* | **Proteobacteria** | 2951 | 10 | 3 | 20 | 21,20 | 0,32 | |
| *Glaciecola* | **Proteobacteria** | 409 | 22 | 3 | 29 | 21,62 | 0,34 | |
| *Mycoplasma* | **Tenericutes** | 1805 | 2 | 8 | 18 | 20,50 | 0,32 | |
| *Pseudoalteromonas* | **Proteobacteria** | 600 | 2 | 9 | 22 | 17,18 | 0,34 | |
| **E) Network for the 50 taxa the most abundant** | | | **Correlations** | | **Topological metrics** | | | |
| **Genus (Inukjuaq)** | **Phylum** | **Abundance** | **Positive** | **Negative** | **DG** | **NC** | | **CC** |
| *Bradyrhizobium* | **Proteobacteria** | 223 | 0 | **1** | 1  *  *  ** | 1  *  *  ** | | 1 |
| *Shewanella* | **Proteobacteria** | 216 | 0 | **1** | 1  *  *  ** | 1  *  *  ** | | 1 |
